# PERGA: A Paired-End Read Guided *De Novo* Assembler for Extending Contigs Using SVM and Look Ahead Approach

**DOI:** 10.1371/journal.pone.0114253

**Published:** 2014-12-02

**Authors:** Xiao Zhu, Henry C. M. Leung, Francis Y. L. Chin, Siu Ming Yiu, Guangri Quan, Bo Liu, Yadong Wang

**Affiliations:** 1 Center for Bioinformatics, School of Computer Science and Technology, Harbin Institute of Technology, Harbin, Heilongjiang, China; 2 Department of Computer Science, University of Hong Kong, Hong Kong; 3 National Pilot School of Software, Harbin Institute of Technology, Weihai, Shandong, China; Harbin Medical University, China

## Abstract

Since the read lengths of high throughput sequencing (HTS) technologies are short, *de novo* assembly which plays significant roles in many applications remains a great challenge. Most of the state-of-the-art approaches base on de Bruijn graph strategy and overlap-layout strategy. However, these approaches which depend on *k*-mers or read overlaps do not fully utilize information of paired-end and single-end reads when resolving branches. Since they treat all single-end reads with overlapped length larger than a fix threshold equally, they fail to use the more confident long overlapped reads for assembling and mix up with the relative short overlapped reads. Moreover, these approaches have not been special designed for handling tandem repeats (repeats occur adjacently in the genome) and they usually break down the contigs near the tandem repeats. We present PERGA (Paired-End Reads Guided Assembler), a novel sequence-reads-guided *de novo* assembly approach, which adopts greedy-like prediction strategy for assembling reads to contigs and scaffolds using paired-end reads and different read overlap size ranging from *O*_max_ to *O*_min_ to resolve the gaps and branches. By constructing a decision model using machine learning approach based on branch features, PERGA can determine the correct extension in 99.7% of cases. When the correct extension cannot be determined, PERGA will try to extend the contig by all feasible extensions and determine the correct extension by using look-ahead approach. Many difficult-resolved branches are due to tandem repeats which are close in the genome. PERGA detects such different copies of the repeats to resolve the branches to make the extension much longer and more accurate. We evaluated PERGA on both Illumina real and simulated datasets ranging from small bacterial genomes to large human chromosome, and it constructed longer and more accurate contigs and scaffolds than other state-of-the-art assemblers. PERGA can be freely downloaded at https://github.com/hitbio/PERGA.

## Introduction

The high throughput sequencing (HTS) technologies have emerged for several years [1], [2] and are widely used in many biomedical applications, such as large scale DNA sequencing [3], re-sequencing [4] and SNP discovery [5], [6], etc. However, since the length of reads generated by HTS technologies (typically 50–150 base pairs [7]–[9]) are much shorter than those of the traditional Sanger sequencing (typically about 800 base pairs [10]), and the per-base sequencing error is high [11], the short read assembly is still a great challenge for genome sequencing.

The overlap-layout strategy and the de Bruijn graph strategy are two major approaches for assembly. The overlap-layout-based approaches firstly compute the overlaps among reads, and then assemble according to the read overlaps, and it consists of the greedy extension strategy and the overlap graph strategy as two subcategories.

The greedy extension approach was applied by first several *de novo* assemblers for the HTS data, such as SSAKE [12], VCAKE [13], SHARCGS [14]. In these assemblers, reads are stored in a prefix/suffix tree to record overlaps, and assembly is performed based on base-by-base 3′ extension according to the simple greedy heuristics of selecting the base with maximum overlap or using the most commonly represented base. In order to prevent mis-assembly, when there are more than one feasible extension due to sequencing errors or similar regions in the genome, the extension will stop. As a result, short contigs will be produced and the genome sequences cannot be reconstructed completely. In many situations, the erroneous extensions (in the multiple feasible extensions) can be detected if the assemblers try to extend for a few bases, e.g. erroneous extensions due to sequencing error at the end of a read usually cannot be extended in later steps (dead ends [15]) and multiple extensions due to sequencing error in the middle of a read should be extended to the same nucleotide in later steps (bubbles [15]). Besides constructing short contigs, these assemblers store the reads and their reverse complements inefficiently, so their memory consumptions are usually very large (especially when there are huge number of erroneous reads with high sequencing depth), which limits their application for large amount of HTS datasets.

To avoid the disadvantage of the greedy extension strategy, Edena [15] and CABOG [16] adopt the overlap graph strategy. This approach constructs an overlap graph in which a vertex represents a unique read and an edge connects vertices *u* and *v* if and only if *u* and *v* overlap each other sufficiently. Assembly is performed by simplifying the graph based on topologies, such as transitive edges, dead ends and bubbles. Each simple path in the simplified graph represents a contig. This approach is also not suitable for HTS data because they require enormous computations to detect overlaps among a great amount of reads. Recently, new methods based on read overlaps using Burrows-Wheeler Transform [17], such as SGA [18] and fermi [5], could assemble larger amount of HTS data. However, they require much more computations to construct a FM-index [19]. The Celera Assembler [20] based assembler MaSuRCA [21] transforms the high coverage data into low coverage but long super-reads to dramatically reduce the overlap computations, which makes it more popular.

The de Bruijn graph strategy, which was firstly introduced in EULER [22], is particularly suitable for short reads of HTS technologies. This strategy can help to reduce the large amount of computations of read overlaps or the construction of FM-index of the overlap-graph approach, and Velvet [23], EULER-SR [24], ALLPATHS [25], ABySS [26], IDBA [27], IDBA-UD [28], SOAPdenovo [29], adopt this strategy. This approach breaks up each read into a collection of overlapping *k*-substrings, called *k*-mers, to construct a de Bruijn graph. In the graph, a vertex represents a unique *k*-mer and an edge connects vertices *u* and *v* if and only if *u* and *v* overlapped by *k*–1 nucleotides and appear consecutively in a read. The graph will then be simplified by removing dead ends and merging bubbles and a simple path in the simplified graph represents a contig. As the *k*-mers have fixed length and erroneous *k*-mers can be detected from their low sampling rates, the de Bruijn graph consumes much less memories than the overlap graph. However, most of them only use a fixed *k*-mer size except IDBA and IDBA-UD. Since small *k* values will lead to better connectivity with much more branches due to repeat segments larger than *k*, whereas large *k* will result in worse connectivity with more gaps due to missing *k*-mers [28]. Most of these assemblers just pick an intermediate *k* to compromise these two problems. IDBA [27] and IDBA-UD [28] give better results by iterating the *k*-mer sizes from *k*_min_ to *k*_max_ by using small *k* to resolve gaps and large *k* to resolve branches.

There are two common problems on the above assemblers.

### 1) Un-fully utilized information of paired-end and single-end reads

Paired-end reads information usually was used for assembling contigs to scaffolds, however, different overlap lengths of paired-end reads and single-end reads usually were not considered when assembling reads to contigs. Thus, some branches that can be resolved using this information become unsolvable. IDBA-UD applies paired-end reads aligned to the same contig for extending the contig (local assembling). However, paired-end and single-end information were considered at equal weight and the number of reads support each branches and the length of overlaps of each supported reads was not considered.

In fact, paired-end reads should be used in the highest priority to resolve branches. Given a branch with two possible extensions (or outgoing edges), one extension is well supported by enough paired-end reads, whereas the other extension has more single-end reads but without well supported paired-end reads, then assembler should extend the contig to the one with more well supported paired-end reads and treat the other as incorrect. If there are no available paired-end reads, single-end reads should be used to determine the correct extension.

Assemblers stop when there is more than one choice for extension without considering the different overlapped lengths supporting each extension. Instead, they usually treat all single-end reads larger than a threshold (or a rate) equally. Given a branch with two possible extensions (or outgoing edges), assembler should extend the contig to the one with more supporting reads and treat the other as incorrect. Even when the numbers of reads supporting both extensions are the same, assembler should extend the contig to the extension with much longer overlaps because short overlapped reads may due to sequencing errors or short repeats.

### 2) Tandem repeats

Because of error in recombination or genome duplication, many repeats are short tandem repeats (e.g., <100 bp) with the occurrence positions of the repeats are close in the genome (e.g. distance of two adjacent occurrences <100 bp) (File S1–S2). These tandem repeats will introduce branches which are difficult to be resolved using the existing assemblers. Assemblers based on overlap-layout strategy stop when there are more than one choice for extension. For de Bruijn graph, these tandem repeats will introduce complicated branches in the de Bruijn graph and existing assemblers cannot correctly separate the different copies of repeats to their correct positions while assembling. For such branches, the existing assemblers usually stop to avoid introducing assembly errors, thus resulting short assembly size.

In order to fully utilize the information in reads for resolving branches in assembling, we introduce PERGA (Paired-End Reads Guided Assembler), a novel *de novo* sequence reads assembler which adopts greedy-like prediction strategy for assembling reads to form contigs and scaffolds. The main contributions of PERGA are as follows.

1. **Utilizing information in paired-end and single-end reads.** Instead of using single-end reads to construct contigs, PERGA uses paired-end reads and different read overlap size thresholds ranging from *O*_max_ to *O*_min_ to resolve the gaps and branches. In PERGA, contigs are extended based on base-by-base extension. Paired-end reads are aligned to contigs for determining possible extensions. When there are not many paired-end reads in some genome regions, single-end reads with variable overlap sizes from larger threshold *O*_max_ to smaller threshold *O*_min_ are applied to handle branches and gaps. Large overlap size *O* **≧** *O*_max_ is used in priority to extend contigs to resolve branches; and if there are missing overlaps for larger *O*, then a degressive smaller *O* will be used to obtain better connectivity to resolve gaps until the read overlap is found before *O* **=** *O*_min_.
2. **SVM navigation model for determining branch.** When there are multiple possible extensions (due to sequencing error or repeats), PERGA will determine which extension is more likely based on branch features, i.e. read coverage levels at the branch site and locally, path weight, gap size (see Materials and Methods). By constructing decision models using machine learning approach (SVM) based on these features, PERGA can determine the correct extension in 99.7% of cases. Note that PERGA will also determine the case and stop extending the contig when both extensions are likely to be correct.
3. **Look ahead approach.** As there are still some mis-predictions (about 0.1∼0.3%), and when the confidence of the prediction is low, PERGA will check all these extensions and determine whether these extensions are due to sequencing errors or repeats. If the multiple extensions are due to sequencing errors (the extensions are similar and converge to the same nucleotide within short distance), PERGA will merge the extensions together to form a single contigs. If the branches were introduced due to short tandem repeats (extensions are different, do not converge and supported by paired-end reads with vary insert distance), PERGA will detect their overlap and separate different copies of repeats to resolve the branches.

In summary, PERGA combines principles of traditional overlap-graph based approaches with novel heuristics for extending a path and resolving the paths at branches. More specifically, it employs four heuristics, from the most conservative to the most relaxed as follows. i) at each point, use compatible paired-end reads to extend the path; ii) if no paired-end reads are available, extend with single-end reads, starting from those with the maximum overlap; iii) for multiple feasible extensions, use a machine learning method (SVM) to distinguish one path, taking into account read coverage levels at the branch site and locally, path weight, gap size; iv) if indistinguishable, employ look-ahead approach to search for possible short stretches of sequencing errors that can be bridged and possible short tandem repeats whose different copies can be separated, before terminating the extension at the branch.

According to our experiments, PERGA gave better performance than other assemblers (Velvet, ABySS, IDBA-UD, CABOG and MaSuRCA) with longer and more accurate contigs (scaffolds) with moderate memory because of its greedy-like prediction model, look-ahead approach and variable overlap size approach to fully utilize the information of paired-end and single-end reads in resolving branches in more accurate way while assembling.

## Materials and Methods

**Figure 1:**
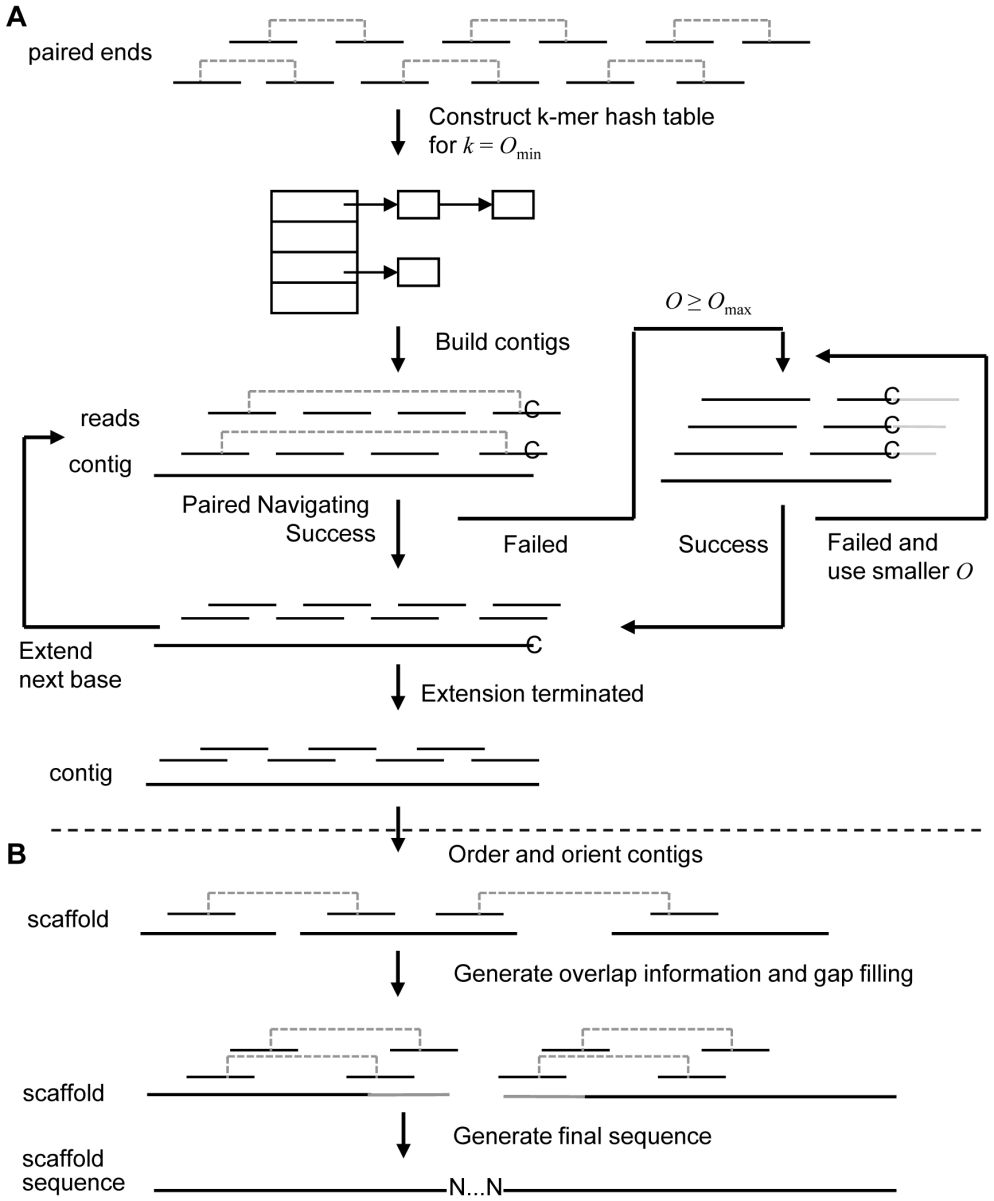
Workflow of PERGA. There are two phases for PERGA. assembly of reads and assembly of contigs**.** (A) Phase 1, assembly of reads. *k*-mer hash table is firstly constructed using paired-end reads for *k* = *O*_min_, then contigs are extended iteratively one base at a time (left feedback loop) at 3′ end by using paired-end reads in high priority, and variable overlap size thresholds ranging from *O*_max_ to *O*_min_ (right feedback loop) if there are no paired-ends. (B) Phase 2, scaffolding. Paired-end reads are used to order and orient contigs, fill intra-scaffold gaps to generate larger scaffolds.

Figure 1 shows the overview of the proposed approach of PERGA, which consists of assembly of reads and assembly of contigs (i.e., scaffolding) as its two phases. In phase 1, a *k-mer hash table*, which is used to represent read overlaps by the consecutive *k*-mers, is constructed from the set of input reads, and then PERGA uses paired-ends and variable read overlap sizes thresholds from *O*_max_ to *O*_min_ to extend contigs. A greedy-like prediction strategy in which a *k*-mer is chosen as a start of contig extension is performed iteratively in the 3′ direction one base at a time until there are either no overlapping reads or a repeat is found, and then the contig will be extended on the 5′ end in the same way. For each base extension, PERGA prefers the reads having more overlaps with contigs and uses the reads having the most represented base for extension. When extending a base, PERGA firstly uses paired-end reads to navigate contig extension with the highest priority, as it can resolve the branches caused by repeats smaller than the insert size with much more confidence than those using single-end reads. However, as there may be genome regions with low sequencing depth and insufficient paired-end reads, PERGA uses single-end reads to extend contigs in such regions by applying the variable overlap size ranging from larger *O*_max_ to smaller *O*_min_ to resolve repeats of sizes smaller than *O*_max_ and to resolve gaps due to the missing large overlaps.

Due to existence of repeats in genome or sequencing errors in reads, there may be branches which have more than one feasible extension with various read occurrences when extending contigs. Instead of stopping the extension, PERGA records the branch information to generate hyperplanes for the paired-ends and single-ends respectively by Support Vector Machine (SVM) method. Finally, these two SVM models are used to determine whether to extend or stop for branches while assembling, and in most cases, branches can be correctly resolved.

However, there are also a few exceptions (branches that are incorrectly stopped or extended) when using the SVM models to decide the navigation. These situations can be resolved by looking ahead to find the feasible paths to resolve the incorrect stops and incorrect extensions. This look-ahead approach can make the contig much longer with fewer mis-assemblies. Note that PERGA determines whether the branches is due to sequencing errors or repeats based on the properties of extended paths and will resolve these two kinds of branches using different methods.

Besides look-ahead approach, PERGA also handles erroneous bases in reads using topological structures, which is similar to the removals of dead ends and bubbles for de Bruijn graph based approaches. During extension, errors at ends of reads will lead to dead ends, and the other errors in the inner part of reads will cause bubbles, PERGA deals with dead ends with lengths smaller than read length and tolerates bubbles with sizes no more than *O*_min_. In PERGA, the dead ends containing erroneous *k*-mers will be excluded from assembly by other correct reads during extension; and the bubbles in reads are deemed as valid substitution.

In phase 2, paired-end reads are aligned onto contigs and are used to order and orient contigs to form scaffolds (i.e., ordered sets of contigs with gaps in between). Then, the overlap sizes and the gap sizes for the linked neighboring contigs are computed, and the overlapped neighboring contigs are merged to form longer contiguous sequences, and the gapped neighboring contigs are processed using a local assembly approach to close their intra-scaffold gaps to generate longer contiguous sequences. Unlike SOAPdenovo [29] which trims *k* bases to exclude erroneous bases at contig ends when scaffolding, PERGA corrects such erroneous bases by pair-wise alignment of the overlapped ends of the neighboring contigs. Finally, the scaffold sequences are generated to form the resultant assembly according to the overlaps and the gap sizes of the contigs in scaffolds.

The details of each step will be described one by one in the following sections.

### Assembly of reads to contigs

The first phase of PERGA is to assemble reads into contigs using a greedy-like prediction method based on paired-end reads information (if possible) and then single-end reads. The algorithm starts with a *k*-mer at the end of an unused read and treats it as contig. PERGA iteratively aligns paired-end reads to contigs and tries to extend it at both ends. In order to determine the possible extension, either A, C, G or T, a SVM model is used to determine whether PERGA should extend the contig using the nucleotide with maximum supports from aligned paired-end reads (instead of extending the contig only when all aligned reads support the same extension as other greedy algorithms) based on the properties of aligned reads. Besides, even when the SVM model cannot determine whether extending the contig or not, PERGA will try to extend the contigs with all possible nucleotides and determine which nucleotide should be used to extend the contig by the later steps (look-ahead approach). After extension, errors in aligned reads can be identified and be corrected for later extension. Details of the assembling step are described as follows.

#### Construct *k*-mer hash table

PERGA applies a *k*-mer based, cost effective approach to perform read alignments. Overlaps of two reads can be represented by their consecutive common *k*-mers, for example, two reads overlap with *w* nucleotides should share *w* – *k* +1 consecutive *k*-mers. Thus, PERGA uses a hash table to store occurrences of *k*-mers in reads. We refer *occurrence* of a *k*-mer as the positions on reads it appears. Note that a *k*-mer may occur in multiple reads and a *k*-mer and its reverse complement are stored at the same entry; and the occurrences of each *k*-mer are stored in an ascending order according to their reads, so that reads can be aligned onto contigs in a fast way. Moreover, in order to reduce the memory consumption, PERGA only samples ten percent of all the *k*-mers of a read at its both ends, and these *k*-mers at read ends are used to align reads to contig in an effect way while assembling.

#### Align reads to contig

Paired-end reads information is used to extend a contig before single-end reads information because it can resolve longer repeats, i.e., up to the insert size of paired-end reads. PERGA automatically infers the mean insert size as well as the standard deviation of the paired-end reads that have been assembled onto contigs. Only those two ends which are aligned in correct directions, i.e. pointed to each other on different strands, are used to extend contigs.

**Figure 2:**
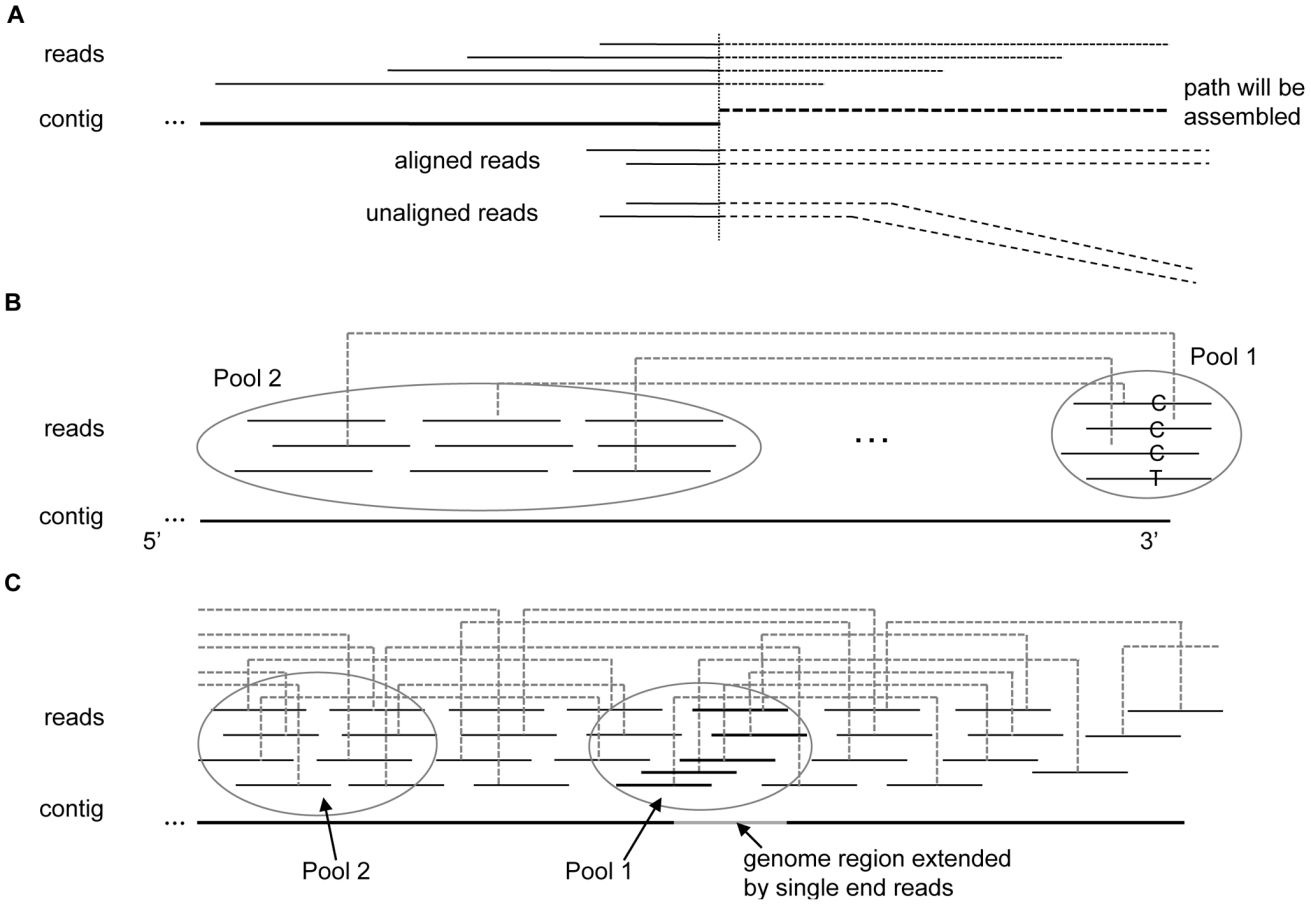
Align reads to contig for extension. (A) Align reads to contig. The path of nearby genome region is extracted according to the reads that are partially aligned onto contig, then new reads having more than 90% aligned bases with the path will be considered correctly aligned; otherwise, they should be aligned onto other genome regions rather than at that position. (B) Extension using paired-end reads. Contig is extended at the 3′ end according to the reads in Pool 1 and whose mates in Pool 2. There are two candidate bases ‘C’ and ‘T’, and ‘C’ is well supported by the mates in the two pools, whereas ‘T’ has no paired-end reads support, thus ‘C’ will be chosen to append onto the contig. (C) Extension using single-end reads. When assemble the grey color region which cannot be assembled by paired-end reads and the reads in Pool 1 have no mates in Pool 2, the reads in Pool 1 are used as single-end reads to extend the contig.

PERGA extracts the paths that will be assembled in near genome region along with assembly, and reads having a large portion of aligned bases (e.g.,>90%) with the paths will be considered to be correctly aligned. Therefore, some reads from other genome regions with only a few aligned bases at the ends will be prevented from the assembly, thus reduce the adverse impact of the short repeats at the reads ends and improve the significance of the correctly aligned reads while assembly, and the prevented reads can be placed to their correct place onto other genome regions (Figure 2A). As shown in Figure 2B, PERGA aligns paired-end reads onto a single contig. Reads with one end totally aligned to the contigs and the other end partially aligned to the contig are used to determine the extension of contig.

When starts the extension of a contig, the *k*-mer at end of a read is selected as the start contig, and the contig is extended iteratively using SVM model to eliminate sequencing errors and avoid the impacts of short repeats by applying the variable overlap size approach based on single-end reads. When the contig is long enough for using paired-end reads, extension will be applied using paired-end reads in the highest priority to avoid repeats shorter than the insert size. Moreover, when the start *k*-mer contains sequencing errors, it typically has low frequency in *k*-mer hash table, and such *k*-mers are excluded from the start construction of a contig. When PERGA cannot determine the extension from the aligned paired-end reads, single-end reads information, including paired-end read with one end aligned to the end of a contig and the other end unaligned, is used to determine the extension of contig (Figure 2C).

#### Utilizing information in paired-end and single-end reads

**Figure 3:**
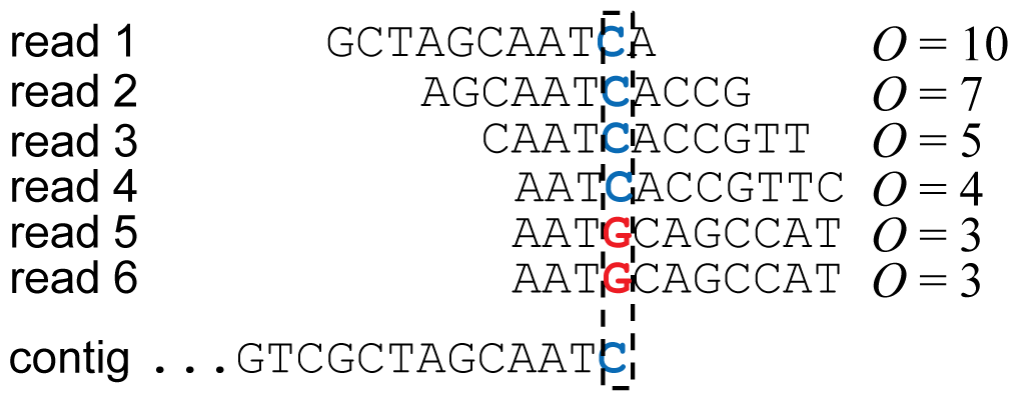
Example of the variable overlap size approach for contig extension. Suppose *k* = *O*_min_ = 3 and *O*_max_ = 6. There are 6 reads, reads 1–4 are the reads that can be assembled onto the contig, while reads 5–6 are the reads that should be assembled onto other regions. The contig is extended using *O* ≥6 by reads 1–2, and if there are no reads having *O* ≥6, then smaller *O* ≥5 will be used in the same way until the contig can be extended successful. AAT is a repeat that can be resolved by *O* ≥4 and GCA is another repeat that has been resolved by reads as such reads do not overlap each other.

Since the alignment of single-end reads are less confident than the paired-end reads especially when the length of aligned region *O* is short, single-end read information is used carefully from reads with large *O* to reads with small *O*. PERGA determines the possible extension using reads with *O* larger than a larger threshold *O*_max_ then to smaller threshold *O*_min_ iteratively. Thus, if PERGA can determine the extension using reads with large *O* confidently, it will not consider those reads with small *O*. In Figure 3, the contig is extended by reads 1 and 2 (*O* ≧6) and resolves the repeat AAT in reads 5 and 6 from other genome regions, and if there are no reads having *O* ≧6, then smaller *O* ≧5 will be applied again in the same way. Moreover, the read overlap approach can resolve repeats in the reads without overlaps among each other, e.g., GCA from reads 1, 2, 5 and 6.

#### SVM navigation model

**Figure 4:**
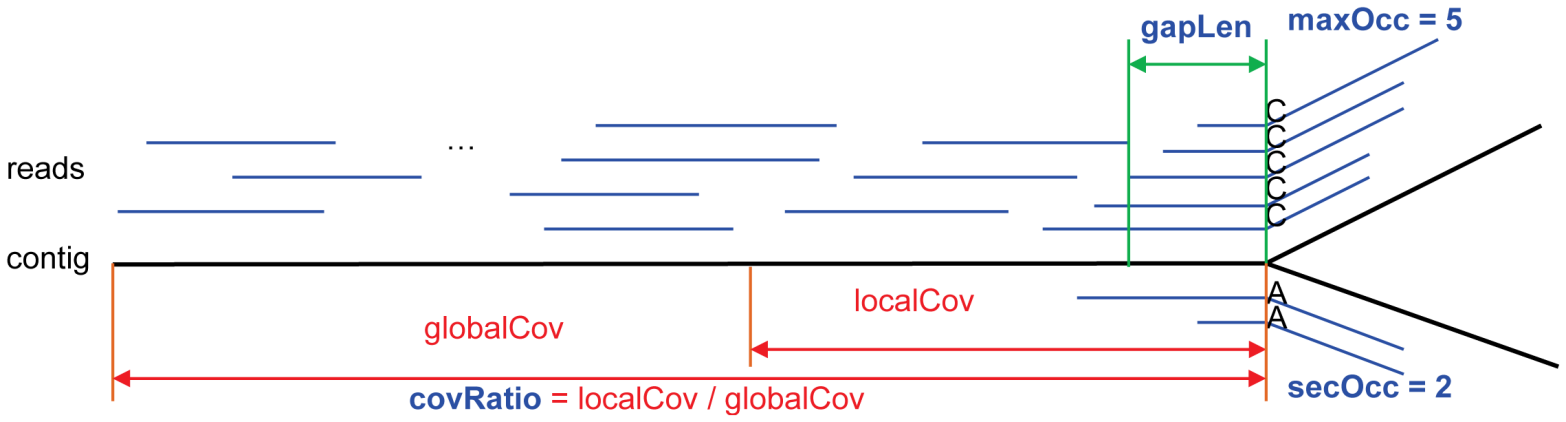
The SVM model to resolve branches. The maxOcc and secOcc should differ as much as possible, the covRatio should be near 1.0 and the gapLen should be as short as possible. Otherwise, contig should be extended more carefully.

When extending contigs, there may be more than one feasible extension with various supporting reads that are mainly due to repeats or sequencing errors, i.e. there is a branch (Figure 4). When determining correct extension at branch, PERGA records the branch information as features (*maxOcc*, *secOcc*, *covRatio*, *gapLen*), where *maxOcc* is the number of reads supporting the majority nucleotide, *secOcc* is the number of reads supporting the second majority nucleotide, *covRatio* is the ratio of the average number of aligned reads (per nucleotide) at the extending ends (within two read lengths) to the average number of aligned reads for the contig, *gapLen* is the distance of the previous completely aligned reads to the contig end. The idea is that for a branch, if its maxOcc and secOcc differ a lot (e.g., secOcc/maxOcc <0.7), the feasible extension corresponding to the maxOcc is usually a correct extension; otherwise, the extension corresponding to maxOcc might be incorrect and should be stopped for further checking. A branch with low gapLen suggests that the number reads aligned to the end of contig is high and the maxOcc should be a correct extension, and a branch with covRatio larger than one suggests that there is a repeat nearby and PERGA should extend more carefully.

For training the SVM prediction models, we recorded the branches of the four features while assembling, and treated each branch as a point in a four-dimensional space in which these points can be used to draw a hyperplane by machine learning approach to separate the branches that should be continued or stopped. By comparing them to the reference while assembling, these branches can be classified into correct extension, wrong extension, correct stop and wrong stop. We used these points as training dataset to train the SVM model, and labelled each branch as CONTINUE if the branch is a correct extension or a wrong stop that should be continued; otherwise it is a STOP branch that should be stopped.

Based on training dataset on branches, PERGA can determine the cases whether we should extend a contig using the majority nucleotide or not. A support vector machine method using polynomial kernel function *K*(*x*, *y*) = <*x*, *y*> ×(1+<*x*, *y*>)^2^, where *x*, *y* are vectors containing branch information, <*x*, *y*> is the dot product of *x*, *y* being constructed based on the four features and is used to determine if a contig should be extended. Note that PERGA will firstly determine the correct extension using features calculated based on aligned paired-end reads. If PERGA fails to decide whether to extend the contig, it will recalculate the features using aligned single-end reads from *O* ≧ *O*_max_ to *O*_min_ and will determine when to extend the contig.

#### Look-ahead approach

Since SVM is not perfect for all of the branches, there are a few low confident cases when using the SVM models to decide whether to extend a contig or not. For these cases, PERGA looks ahead to find all feasible extensions. Starting from the branches, PERGA extracts all paths that will be appended to contig using the reads, and compares the sequences of these paths. Based on the assumption that if the multiple extensions are due to repeats, it will be hard to get a highly agreed consensus sequence than the case that the multiple extensions are due to sequencing errors, PERGA calculates the ratio of the majority nucleotide at each position and assumes the majority nucleotide is incorrect if its ratio is less than 0.9. If there are less than 3 incorrect nucleotides, the extension will continue using the majority nucleotide, otherwise, PERGA will determine if the branches is due to short tandem repeats.

#### Deal with short tandem repeats

**Figure 5:**
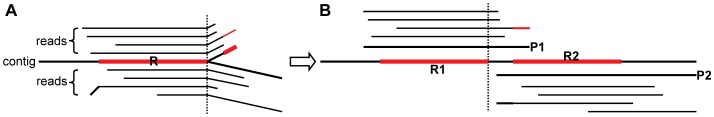
The approach to separate repeat R to its two copies R1 and R2. (A) The branch is caused by different copies of repeat R (bold red). (B) The two copies of repeat R are resolved by two paths P1 and P2. P1 and P2 are extracted according to the reads at that branch, and the suffix of P1 overlaps the prefix of P2, then the reads of P2 are adjusted to their correct positions.

As our observations, there are some short repeats with lengths less than the read length, and some of them have a short distance, e.g., less than a read (File S1–S2). Such repeats may cause ambiguities while assembling, thus should be resolved for better accuracy. If the path overlap is significant (e.g.,>10 bp) (e.g., path P1 and P2 overlaps in Figure 5), which usually means that there is another repeat in nearby genome region that will be assembled recently, and the two paths can be merged and the aligned reads on path P2 will be adjusted according to the overlap, and the extension will continue according to the path (e.g., P1) at the branch; otherwise, It usually means that these repeat copies are from different genome regions with a large distance, and the extension should stop to prevent assembly errors.

When there are more than one repeat after merging overlapped paths, PERGA firstly calculates the mismatched base count of each path comparing to contig, and then computes the distance of the paired ends of each path that one end aligned on the path and the other end aligned on contig. For each path, if its mismatched base count is significant (e.g.,>2) and its distance is much different from the insert distance (e.g., difference>2 * standard deviation of insert distance), which usually means that the repeat copy (i.e., the path) may come from other genome regions in high probability rather than from the branch, so such paths will be invalid and be removed together with their aligned reads, and these removed reads can be used for later assembly of other genome regions.

#### Handling erroneous bases

**Figure 6:**
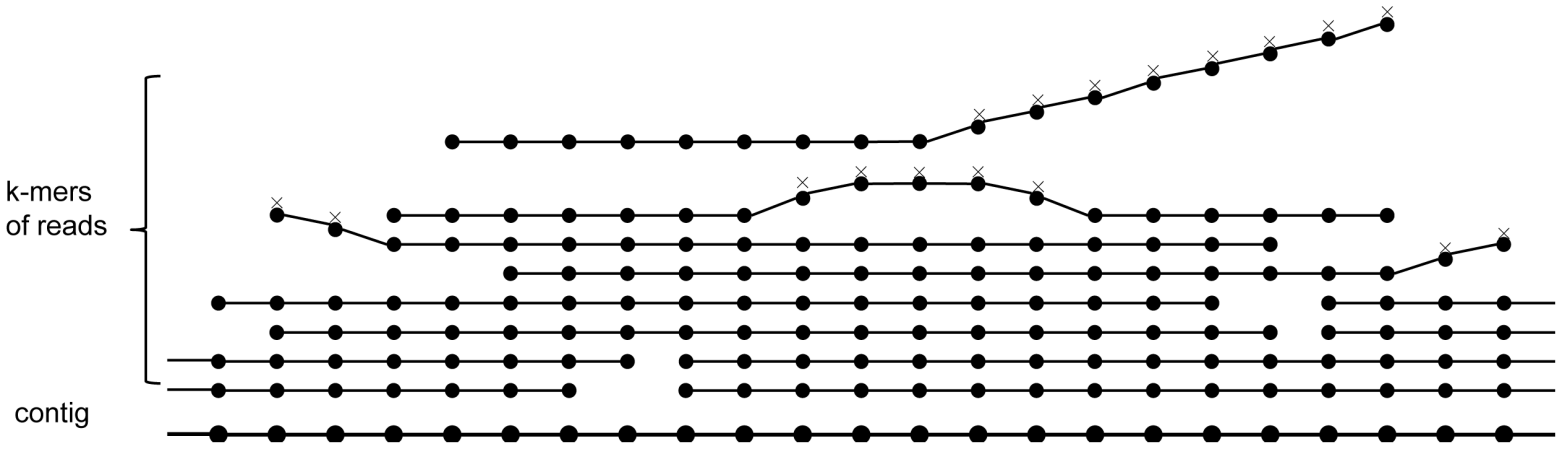
Scheme for removing erroneous bases. Erroneous bases in reads will cause dead ends and bubbles that can be implicitly resolved as these errors can be masked by these correct reads. Reads with low similarities probably can be assembled onto other contigs with higher similarity.

Erroneous bases in reads for HTS data can make the assembly problem much more complex and error-prone, and cannot be easily solved by paired-end reads and variable overlap size approach. To resolve ambiguities arising due to sequencing errors, PERGA applies a method similar to the approach based on topological structures [15], [23], [26]. Errors at the ends of reads usually lead to short dead ends which are likely to be terminated prematurely, and errors in the inner part of reads will cause small bubbles in which the two paths have similar bases with the same starting and ending reads (Figure 6). Note that such dead ends and bubbles are checked in reads rather than in contigs, and PERGA checks such errors and corrects them for later extensions. It checks the similarity between a read and the contig according to topological structures, and if similarity is high, say ≧95%, then the read is assembled onto contigs; otherwise, the read will be not assembled onto contigs, instead, it might be assembled into other genome regions with higher similarity.

### Assembly of contigs

PERGA assembles generated contigs into larger scaffolds using paired-end information similar as existing assemblers. In this procedure, reads are aligned onto contig ends to order and orient contigs to generate scaffolds. After constructing scaffolds, PERGA merges the overlapped neighboring contigs, fills intra-scaffold gaps, and generates consensus sequences to give final assembly (Figure 1B). Detailed scaffolding method is described in the following subsections.

#### Reads alignment

If one end of a paired-end read uniquely aligned onto one contig and the other end uniquely aligned onto another contig, these two contigs should appear adjacently in the genome. Note that reads aligned to multiple contigs should not be considered. As reads with both ends aligned to the same contigs does not provide extra information for constructing scaffold, PERGA aligns reads to the end of contig, called *linking region*, which has 2 kbp by default.

#### Linking contigs to scaffolds

**Figure 7:**
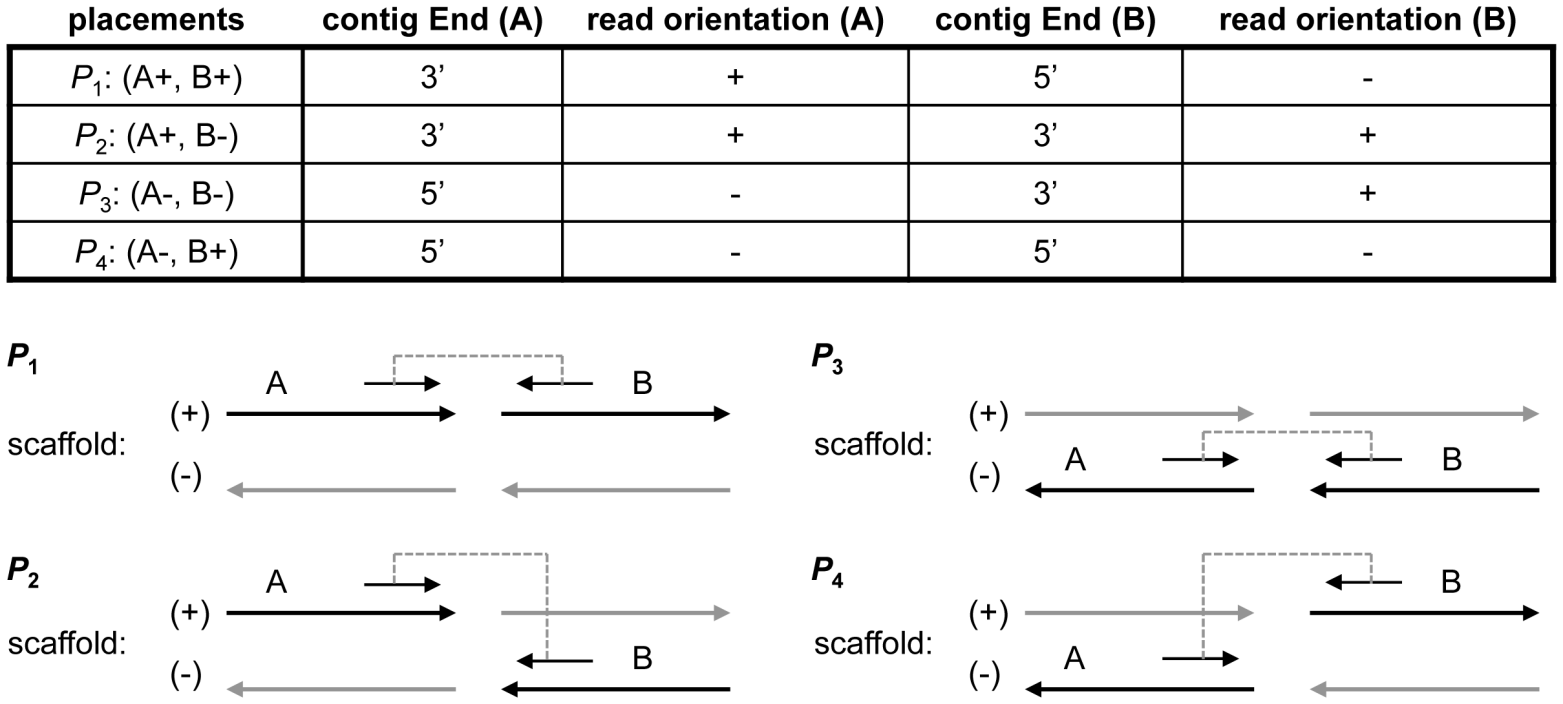
Four placements for two adjacent contigs. Placements depicted at bottom correspond to the ones in top table. Adjacent contigs (bold arrows) are placed based on their aligned read pairs. Grey arrows indicate reverse complements of contigs. Contig orientation (‘+’/‘−’) in top table is the contig orientation in scaffolds.

Since reads may be sampled from positive strand or negative strand randomly and whether a contig sequence represents the positive strand or negative strand is unknown, there are four valid *placements* 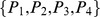 for two adjacent contigs (A, B) as shown in Figure 7. The relative positions and directions of the contigs can be determined from the aligned paired-end reads. However, because of sequencing errors and misalignment, the relative direction and position of two contigs can be different using different paired-end reads. In order to determine the correct relative direction and position of contigs, a *scaffold graph* 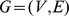 is constructed over the set of linking regions 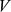 to capture all placements of adjacent contigs by the set of edges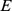. In the graph, *placement weight* is defined as the number of paired-end reads support each placement of two linking regions 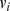 and 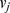 in distinct contigs, and each edge 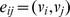 is associated with a quaternion 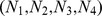, where 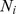 is the weight for placement 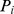. Only uniquely aligned reads are used to construct graph to prevent introducing errors by repeats.

**Figure 8:**
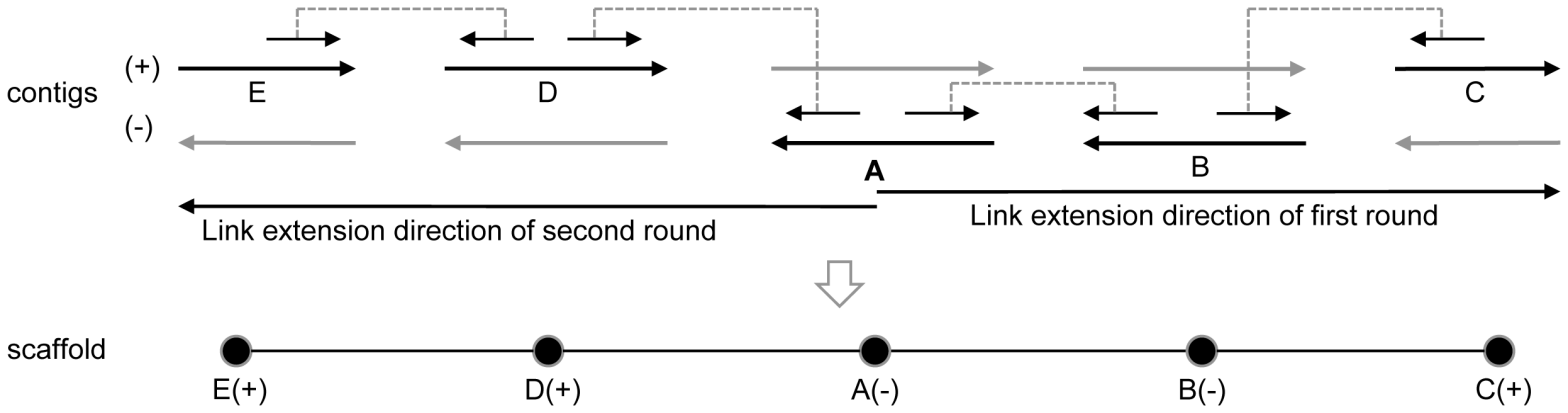
Scheme for contigs linking. The first link round (right) extends contigs by paired-end reads from the starting contig A to the right until no extension are possible, then the second link round (left) is carried out from A to the left in the same way. Scaffold is a linear structure of a set of linked contigs (bottom) that have been ordered and oriented.

Contigs are linked based on a greedy approach. A contig longer than the linking region size is randomly selected as the initial scaffold to be extended. The extension is performed iteratively by including the neighboring contigs to the right, and once a contig is included in a scaffold, its orientation is assigned according to the placement. Extension is performed iteratively and is terminated until no neighboring contigs or multiple candidates undifferentiated which one is correct. When the extension is terminated from the 3′ end, the 5′ end will be extended in a similar fashion (Figure 8). After contigs are linked, their orders and orientations in scaffolds will be determined.

#### Overlap between contigs

To generate final scaffolds, it is necessary to compute the distance between each two adjacent contigs in scaffolds, which may be overlapped or have gaps in between. For overlapped contigs, the overlapped region will be detected and the two contigs should be merged into a single contig. For contigs with gaps in between, the gap size will be computed according to the paired-end reads that link the two contigs.

PERGA firstly estimates the gap size between adjacent contigs in scaffolds. Given the paired-end reads with ends *a* and *b* aligned to different contigs A and B, respectively, the gap size *g* can be estimated by 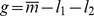, where 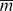 is the mean insert size, 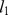 is the distance from 5′ end of read *a* to the gap margin of contig A, 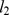 is the distance from 5′ end of read *b* to gap margin of contig B. In practice, there can be multiple such paired-ends aligned onto two adjacent contigs, thus the final gap size 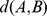 can be inferred by
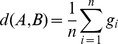
where 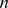 is the number of read pairs between contigs A and B.

PERGA further checks the inferred gap size. If the inferred gap size is a large positive number, there is probably a gap between contigs with the estimated gap size; and if the gap size is a large negative number, there is probably an overlap. If the gap size is not significant, further check is needed by comparing the prefix and suffix of the two contigs.

When the gap size is a large negative number or insignificant, the two contigs may overlap with certain proportion. Because of sequencing error and mistakes in assembling, the overlapping sequence may not be exactly the same. PERGA performs the pair-wise sequence alignment to capture the overlaps. If an overlap is larger than 3 and is agreed with the estimated gap size, this pair of contigs will be recorded as overlapping contigs and merged into a single contig; otherwise, there will be a gap between them.

#### Gap filling

After estimating gap size between adjacent contigs, it is necessary to fill the gap regions for better continuity by local assembly using paired-end reads with one end aligned onto contigs and the other end aligned in gap regions. Most of the sequences in gap regions are repetitive sequences, thus gap filling can be used to resolve such repeats. As the sequences adjacent to gap regions have been recognized, repetitive sequences in gap regions can be easily reconstructed by local assembly which is based on the algorithm of assembly of reads for PERGA using paired-ends.

Consensus sequences are generated from contigs in scaffolds considering their overlaps and gaps. If adjacent contigs are overlapped, then they will be merged; and if contigs are gapped, the gap region between these contigs will be filled with ambiguous bases (‘N’).

### Datasets

We evaluated the performances of PERGA on both simulated and real datasets of Escherichia coli, Schizosaccharomyces pombe and human chromosome 14 (details are shown in Table 1) with reference sizes ranging from 4.6 Mbp to 88.3 Mbp (million base pairs). The simulated Illumina paired-ends datasets were generated using GemSIM [30] with various coverages 50x, 60x, 100x for *E.coli* (can be downloaded from https://github.com/hitbio/PERGA), 50x for *S.pombe*, 50x for human chromosome 14; and the real Illumina *E.coli* paired-end reads data were downloaded from http://bix.ucsd.edu/projects/singlecell/nbt_data.html, with standard genomic DNA prepared from culture, with coverage around 600x, the real *S.pombe* data were downloaded from NCBI (SRA accession: ERX174934), the real human chromosome 14 data were downloaded from http://gage.cbcb.umd.edu/data, this dataset had already been error-corrected using Quake [31] by Salzberg et al [32]. We evaluated the performance of PERGA on resolving branches using SVM prediction model and look-ahead approach. We also compared the performance of PERGA in assembling with other leading state-of-the-art assemblers, including IDBA-UD (v1.0.9) [28], ABySS (v1.3.2) [26], Velvet (v1.2.01) [23], and overlap-based assemblers SGA (v0.9.20) [18], CABOG (v7.0) [16] and MaSuRCA (v2.2.1) [21].

**Table 1:** Datasets D1∼D8 for assemblies.

Datasets	D1	D2	D3	D4	D5	D6	D7	D8
Organism	*E.coli* K12 MG1655				*S.pombe* 972 h-		Human chr14	
Ref. size	4.64 Mbp				12.59 Mbp		88.29 Mbp	
Data type	simulated	simulated	simulated	real	simulated	real	simulated	real
Read length	100 bp	100 bp	100 bp	100 bp	100 bp	100 bp	100 bp	101 bp
#Reads (million)	2×1.16	2×1.4	2×2.3	2×14.2	2×3.1	2×3.3	2×22.1	2×16.3
Cov. depth	50×	60×	100×	600×	50×	52×	50×	40×
Insert size (bp)	370±56	370±58	366±59	215±11	370±57	380±82	366±49	158±17

The RefSeq for *E.coli* K12 MG 1655 is NC_000913.2; the RefSeq for *S.pombe* 972 h- are NC_003424.3, NC_003423.3, NC_003421.2, NC_001326.1; the refSeq for human chromosome 14 is NT_026437.12.

To evaluate the performances of each assembler, we used the length of N50 to evaluate their length metrics, and we used BLASTN (v2.2.25+) [33] to align the contigs and scaffolds to reference to evaluate their accuracy by using reference covered ratio, number and lengths of mis-assemblies. If a contig (or scaffold) entirely matches with the reference with similarity <95%, it is considered as a mis-assembled contigs. As Velvet is a scaffold-only assembler for paired-ends data, we split the scaffolds at the positions of poly-N to get the contigs for comparisons. Note that repeats from different genomic regions will be collapsed into a single copy which can be aligned to more than one location or in disjoint locations when using BLASTN, and we also deem that all those genomic locations are covered by these repeats.

We tested the performance of SVM approach as well as the look ahead approach first. The results were shown in Tables 2–3. And then, we compared the performance of PERGA to other leading state-of-the-art assemblers, the main items of the results were listed in Tables 4–11 with details in Tables S1–S8 in File S1.

**Table 2:** Statistical results for greedy-like prediction model.

Datasets	Correct extensions	Incorrect extensions	Correct stops	Incorrect stops
D1	70299 (99.70%)	60 (0.09%)	123 (0.17%)	26 (0.04%)
D2	84829 (99.74%)	46 (0.05%)	148 (0.18%)	25 (0.03%)
D3	136309 (99.82%)	48 (0.04%)	169 (0.12%)	27 (0.02%)

**Table 3:** Statistical results for look-ahead approach.

	checking sequencing errors			checking short repeats			Overall
Datasets	Correct navi.	Incorrect navi.	Sum	Correct navi.	Incorrect navi.	Sum	Correct navi.
D1	448 (97.60%)	11 (2.40%)	459	174 (98.31%)	3 (1.69%)	177	445 (99.3%)
D2	443 (98.44%)	7 (1.56%)	450	174 (99.43%)	1 (0.57%)	175	442 (99.8%)
D3	485 (98.98%)	5 (1.02%)	490	223 (98.24%)	4 (1.76%)	227	481 (99.2%)

**Table 4:** Evaluation for *E.coli* simulated short reads data (D1, 50×).

		Contigs			Scaffolds				
	*k*/*O*	N50 (kbp)	Cov. (%)	Misass. (#/sum)	N50 (kbp)	Cov. (%)	Misass. (#/sum)	Time (min)	Mem. (GB)
PERGA	*O*≧25	**174.7**	**100.0**	**0**	**174.7**	**100.0**	**0**	**3**	0.9
IDBA-UD	default	112.6	99.98	2/559	148.5	99.98	1/321	11	**0.6**
ABySS	*k* = 45	119.2	99.90	**0**	119.2	99.42	1/3617	9	1.0
Velvet	*k* = 45	108.1	99.76	7/6658	148.3	99.89	1/1596	**3**	0.9
SGA	*O*≧31	24.1	98.57	**0**	95.5	98.59	1/4120	43	**0.6**
CABOG	default	83.1	99.03	1/2638	88.5	99.03	1/2638	77	2.6
MaSuRCA	default	172.8	87.98	4/560k	172.8	87.98	4/560k	16	2.2

**Table 5:** Evaluation for *E.coli* simulated short reads data (D2, 60×).

		Contigs			Scaffolds				
	*k*/*O*	N50 (kbp)	Cov. (%)	Misass. (#/sum)	N50 (kbp)	Cov. (%)	Misass. (#/sum)	Time (min)	Mem. (GB)
PERGA	*O*≧25	**173.9**	**99.99**	**0**	**173.9**	**99.99**	**0**	**3**	1.0
IDBA-UD	default	124.6	**99.99**	**0**	**173.9**	99.97	**0**	13	**0.6**
ABySS	*k* = 45	119.2	99.92	**0**	135.0	99.56	1/25k	10	1.1
Velvet	*k* = 45	125.2	99.79	6/4451	148.5	99.87	**0**	5	1.0
SGA	*O*≧31	23.5	98.35	**0**	95.4	98.48	1/492	50	**0.6**
CABOG	default	68.4	98.72	**0**	77.1	98.64	1/4996	98	2.6
MaSuRCA	default	156.4	94.25	2/257k	156.4	94.25	2/257k	19	2.2

**Table 6:** Evaluation for *E.coli* simulated short reads data (D3, 100×).

		Contigs			Scaffolds				
	*k*/*O*	N50 (kbp)	Cov. (%)	Misass. (#/sum)	N50 (kbp)	Cov. (%)	Misass. (#/sum)	Time (min)	Mem. (GB)
PERGA	*O*≧25	**174.7**	**99.99**	**0**	**174.7**	99.99	**0**	**5**	1.2
IDBA-UD	default	124.6	**99.99**	**0**	148.6	99.96	2/1723	21	0.7
ABySS	*k* = 45	126.2	99.90	1/524	135.0	90.89	4/206k	16	1.7
Velvet	*k* = 45	117.5	99.75	9/7347	148.5	**100.0**	**0**	7	1.4
SGA	*O*≧31	21.7	98.16	**0**	105.6	98.46	2/1024	103	**0.6**
CABOG	default	37.3	93.63	1/61k	56.7	93.56	1/65k	209	2.6
MaSuRCA	default	148.8	98.89	2/54k	172.2	92.15	4/371k	29	2.4

**Table 7:** Evaluation for *E.coli* real short reads data (D4, 600×).

		Contigs			Scaffolds				
	*k*/*O*	N50 (kbp)	Cov. (%)	Misass. (#/sum)	N50 (kbp)	Cov. (%)	Misass. (#/sum)	Time (min)	Mem. (GB)
PERGA	*O*≧25	**133.5**	**99.99**	1/207	**154.8**	**99.99**	1/207	**21**	3.8
IDBA-UD	default	106.8	99.93	1/2105	148.5	99.98	**0**	31	2.0
ABySS	*k* = 45	96.0	93.61	4/293k	113.4	91.45	5/372k	64	**0.3**
Velvet	*k* = 45	82.8	95.25	11/212k	95.5	86.21	5/633k	33	5.1
SGA	*O*≧31	19.3	98.06	**0**	21.3	98.15	1/411	357	5.9
CABOG	Could not be run correctly as it required lots of disk space that exceeded our machine								
MaSuRCA	default	72.3	97.33	6/126k	77.6	97.29	7/129k	118	2.5

**Table 8:** Evaluation for *S.pombe* simulated short reads data (D5, 50×).

		Contigs			Scaffolds				
	*k*/*O*	N50 (kbp)	Cov. (%)	Misass. (#/sum)	N50 (kbp)	Cov. (%)	Misass. (#/sum)	Time (min)	Mem. (GB)
PERGA	*O*≧25	255.4	99.91	**0**	386.7	**99.90**	**0**	**8**	1.7
IDBA-UD	default	137.7	**99.99**	3/966	254.7	99.08	6/100k	31	**1.3**
ABySS	*k* = 45	181.8	99.78	12/9k	211.0	76.72	21/2.7M	25	1.6
Velvet	*k* = 45	158.6	99.74	15/6k	293.2	99.74	8/8k	11	1.9
SGA	*O*≧31	43.0	98.12	1/214	155.1	98.95	4/27k	103	2.0
CABOG	default	139.6	95.24	3/218k	157.1	90.88	6/778k	243	2.5
MaSuRCA	default	**417.9**	90.76	6/1.3M	**417.9**	90.76	6/1.3M	43	2.7

**Table 9:** Evaluation for *S.pombe* real short reads data (D6, 52×).

		Contigs			Scaffolds				
	*k*/*O*	N50 (kbp)	Cov. (%)	Misass. (#/sum)	N50 (kbp)	Cov. (%)	Misass. (#/sum)	Time (min)	Mem. (GB)
PERGA	*O*≧25	**37.0**	**98.97**	17/71k	**70.3**	**98.97**	**17/73k**	7	1.7
IDBA-UD	default	32.1	98.54	28/140k	54.0	97.56	35/247k	31	1.3
ABySS	*k* = 45	33.3	98.20	44/73k	35.7	96.29	48/230k	21	**0.8**
Velvet	*k* = 45	28.7	97.36	26/175k	42.3	95.86	29/391k	**6**	3.9
SGA	*O*≧31	21.3	97.10	**16/38k**	39.1	97.32	21/100k	114	2.0
CABOG	default	22.3	95.12	8/71k	49.4	98.95	11/396k	705	7.3
MaSuRCA	default	36.2	97.0	17/210k	64.7	93.71	20/672k	70	2.7

**Table 10:** Evaluation for human chromosome 14 simulated short reads data (D7, 50×).

		Contigs			Scaffolds				
	*k*/*O*	N50 (kbp)	Cov. (%)	Misass. (#/sum)	N50 (kbp)	Cov. (%)	Misass. (#/sum)	Time (min)	Mem. (GB)
PERGA	*O*≧25	**149.9**	99.54	22/60k	**229.5**	**99.58**	**21/30k**	169	9.3
IDBA-UD	default	66.7	**99.74**	44/152k	174.3	98.51	58/1.24M	**144**	8.7
ABySS	*k* = 45	11.4	94.98	377/354k	30.2	83.40	1109/11M	331	**6.0**
Velvet	*k* = 45	8.6	92.24	1642/3.4M	78.6	24.87	1655/67M	147	13.5
SGA	*O*≧31	2.7	85.36	**146/43k**	5.5	80.10	1980/5M	1360	17
CABOG	default	69.3	76.43	285/19M	82.8	67.40	318/26.6M	2742	11
MaSuRCA	Could not be run correctly because of unknown running error								

**Table 11:** Evaluation for human chromosome 14 real short reads data (D8, 40×).

		Contigs			Scaffolds				
	*k*/*O*	N50 (kbp)	Cov. (%)	Misass. (#/sum)	N50 (kbp)	Cov. (%)	Misass. (#/sum)	Time (min)	Mem. (GB)
PERGA	*O*≧25	11.8	**95.86**	435/2.8M	20.2	91.96	423/6.1M	194	7.9
IDBA-UD	default	**16.3**	94.81	351/4.1M	**21.8**	92.94	335/5.8M	122	8.0
ABySS	*k* = 45	3.9	92.06	485/574k	4.1	91.61	547/975k	161	6.4
Velvet	*k* = 45	3.8	89.62	1199/2.8M	6.6	70.44	4342/21M	**68**	6.5
SGA	*O*≧31	2.4	84.36	**255/95k**	2.7	84.21	247/1.5M	826	16
CABOG	default	13.0	87.65	527/7.3M	20.6	82.07	560/13M	1757	10
MaSuRCA	default	6.8	95.38	165/616k	6.9	**95.28**	**170/710k**	478	**2.7**

The experiments for the simulated reads data were carried out on a 64-bit Linux machine with an Intel(R) Core-2 CPU 2.53-GHz supplied with 3 GB memory except the experiments for CABOG. The experiments for CABOG and the real reads data were carried out on an Intel(R) Xeon(R) Core-8 CPU 2.00-GHz server supplied with 24 GB memory.

## Results and Discussion

### Performance of greedy-like prediction model

The performance of our greedy-like SVM prediction model was assessed by counting the numbers of correctly and incorrectly predicted extensions and stops for all branches during the assembling step. To evaluate the greedy-like prediction model independently, statistics were calculated when the look-ahead approach was not used. The statistical results are shown in Table 2. By constructing the decision models using machine learning approach, PERGA can determine the correct extension in 99.7% of cases for the simulated reads data D1∼D3. And PERGA also determines the stop cases that both extensions are likely to be correct. PERGA can produce only a few incorrect extensions and incorrect stops (less than 0.1%).

### Performance of look-ahead approach

Although the performance of SVM prediction model is good, PERGA still has some incorrect extensions and stops with low confidence. These low confident predictions can be resolved by the look-ahead approach. Table 3 shows the number of correct and incorrect navigations for low confident branches when applying this approach to resolve branches due to sequencing errors and short tandem repeats on the datasets D1∼D3. The statistics for sequencing errors were calculated independently without considering short repeats; and for the low confident branches with unsatisfied properties due to sequencing errors, further check was applied to determine if these branches were due to short tandem repeat.

For the branches due to sequencing errors, most of them can be correctly resolved in high probability (about 98%) with low error rate (about 2%), which makes PERGA generates long and accurate contigs. According to Table 2 and Table 3, less than 1% of the branches (D1∼D3) are adjusted by this approach. As look-ahead approach is very effective, the mis-prediction branches can be easily handled by this approach.

From the table, it can be seen that about one third of the branches do not satisfy the properties for branches due to sequencing error. These branches will be further checked according to the properties for branches due to short repeats. Among these branches, there were only a few incorrect navigations (<2%). Thus, the overall correct navigations for look-ahead approach, after dealing with sequencing errors and short tandem repeats, were more than 99% of cases.

In summary, the greedy-prediction navigation model resolved most (>99%) of the branches, and then the look-ahead approach further resolved most (>99%) of the low confident branches. Therefore, after combining the greedy-like prediction model and look-ahead approach, PERGA can produce long and accurate contigs.

### Performance on *E.coli* genome

Table 4 shows the performances of PERGA as well as other assemblers on the 50x simulated paired-end reads dataset D1. PERGA generated the longest contigs in N50 measures, highest reference coverage and the most accurate result with no mis-assemblies. PERGA and Velvet were the fastest assemblers among seven assemblers with moderate memory usage and were about 3 times faster than IDBA-UD and ABySS, since SGA uses the FM-index to compute the read overlap, and CABOG computes the read overlaps between each other, thus they cost more time while assembling (43 minutes and 77 minutes). MaSuRCA generated 4 mis-assembled contigs (560 kbp) while PERGA did not. This is because that PERGA handles branches for extension much more carefully, it utilizes the greedy-like prediction SVM models which contains much branch information to give much better extensions, and PERGA distinguishes sequencing errors and repeats for branches using the look-ahead approach to decide the correct extensions. Thus, PERGA can provide fewer but longer contigs than the others without producing erroneous contigs.

When the number of reads in the dataset increases, the running times of all the other assemblers increase (Table 5 for dataset D2). However, the running time of PERGA does not increase significantly and is still the fastest assembler. The contigs and scaffolds produced by PERGA have the largest N50 and coverage with no mis-assemblies. Because of the increase in sequencing depth, IDBA-UD and Velvet performed better than on D2 with contig N50 increased from 112.6 kbp and 108.1 kbp to 124.6 kbp and 125.2 kbp respectively with less assembly errors, whereas MaSuRCA had lower N50 size than on D1. PERGA and IDBA-UD had the largest scaffold sizes (173.9 kbp), whereas the results of other assemblers were much shorter (only around 140 kbp). MaSuRCA tended to produce more erroneous contigs and scaffolds, which decreased its genome coverage. Compared with other assemblers, SGA and CABOG produced shorter contigs and scaffolds with longer running times. The coverage of all assemblers on D1 and D2 are much the same and they do not differ much between contigs construction and scaffolds production.

For the simulated 100x dataset D3 (Table 6), since the sequencing depth is high, all assemblers generated similar numbers of contigs with similar coverage in both contigs and scaffolds except ABySS and MaSuRCA which dropped from 99.90% to 90.89% and from 98.89% to 92.15% because of the mis-assembled 4 scaffolds (206 kbp) and mis-assembled 4 scaffolds (371 kbp) for ABySS and MaSuRCA, respectively. MaSuRCA had the most erroneous scaffolds, CABOG generated 1 mis-assembled contig (61 kbp) and 1 mis-assembled scaffold (65 kbp), thus its genome coverage dropped to 93.5%. From the experiments on D1∼D3, it can be observed that CABOG may be not suitable for high coverage data since its contigs (scaffolds) sizes decreased with the increasing coverage depth, and it can also be seen that the overlap-based assemblers (SGA and CABOG) is not suitable for high coverage data. PERGA also was the fastest assembler and produced the most accurate results while others all produced several mis-assembled contigs (scaffolds). In all experiments on simulated data, PERGA did not produce any mis-assembled contigs and scaffolds while the other assemblers mis-assembled some reads in some datasets.

We further used the downloaded *E.coli* dataset D4 with coverage ∼600x to highlight the performance of PERGA on high coverage data, and compared its performances with other assemblers. CABOG could not be run on D4 because it required lots of disk space that exceeded the server. Before assembling, paired-end reads data were corrected using Quake [31], and the results were shown in Table 7.

The overall performance of all other assemblers dropped because of the short insert size dropped from 370 bp to 215 bp, and some repeats with length falling in this range could not be resolved. PERGA still had the best performance in N50 size, maximal size, and genome coverage. It may suggest that the SVM model used by PERGA can capture the properties in real datasets. PERGA was the fastest assembler and generated the longest contigs (scaffolds) (133.5 kbp and 154.8 kbp) with the highest coverage (99.99%), while others assemblers had much lower N50 except scaffolds of IDBA-UD (148.5 kbp). Since PERGA generated very long and accurate contigs, the scaffolds produced by PERGA had the largest N50 and highest coverage even though it did not connect many contigs in scaffolding. As the sequencing depth increased from 50x to 600x, the contigs (scaffolds) N50 size of MaSuRCA decreased from 172 kbp to 77 kbp and the number of contigs (scaffolds) increased from 70 to 240, which may indicate that MaSuRCA is not suitable for high coverage datasets.

After scaffolding, ABySS and Velvet produced longer scaffolds with lower coverage, while PERGA and IDBA-UD did not have coverage difference between contigs and scaffolds as they produced accurate assemblies. ABySS and Velvet both had>200 kbp mis-assembled contigs and >300 kbp mis-assembled scaffolds, thus their contig coverage dropped dramatically from 96% to 86%, MaSuRCA also generated a few erroneous contigs and scaffolds (>100 kbp), and SGA generated accurate contigs and scaffolds, however, its N50 sizes are very small (19.3 kbp and 21.3 kbp), and it used more time than others. This shows that ABySS, Velvet, SGA and MaSuRCA might not be suitable for high coverage sequencing data. They can have good performance on low coverage data but might not be good on high coverage data.

### Performance on *S.pombe* genome

We also tested the performance of PERGA on *S.pombe* 50x simulated dataset D5 and 52x real dataset D6, and the reads of real *S.pombe* dataset were error-corrected using Quake [31] prior to assembly, the results were shown in Tables 8–9.

From Table 8, MaSuRCA, PERGA and Velvet were the top three assemblers in scaffold N50 size (417.9 kbp, 386.7 kbp and 293.2 kbp), whereas the scaffold N50 size of other assemblers were all less than 300 kbp with a few assembly errors, and however, the genome coverage of MaSuRCA was only 90.76% because of its 6 mis-assembled large scaffolds (1.3 Mbp). ABySS generated more mis-assembled scaffolds than others (2.7 Mbp), so its genome coverage dropped from 99.78% to 76.7%. IDBA-UD generated the short contigs and fewer mis-assemblies than ABySS and Velvet, and its scaffolds have more errors than Velvet. SGA had the most number but shortest contigs and scaffolds (N50 size 43.0 kbp and 155.1 kbp), and CABOG generated short scaffolds (N50 size 157.1 kbp) with a few errors (778 kbp). Overall, PERGA outperformed other assemblers on D5 in assembly length, accuracy, coverage and running time.

The results for *S.pombe* real dataset D6 were listed in Table 9. From the table, all assemblers produced similar results in terms of length, accuracy and coverage. PERGA generated the largest contigs and scaffolds (N50 size 37.0 kbp and 70.3 kbp) with highest genome coverage (98.97%) and fewer assembly errors (73 kbp). PERGA, MaSuRCA and IDBA-UD were the top three assemblers in scaffold N50 size (>54 kbp), whereas the N50 size of others were less than 50 kbp. PERGA and IDBA-UD generated the largest scaffold size (334.7 kbp and 270.3 kbp), and MaSuRCA was more error prone and more likely to produce erroneous contigs and scaffolds (210 kbp and 672 kbp). Velvet and PERGA were much faster than other assemblers; however, Velvet produced more errors (175 kbp and 239 kbp) with a high memory cost (3.9 GB). SGA and CABOG needed more time than others, and CABOG required the most time and memory consumption among all the assemblers, whereas ABySS had the least memory consumption (0.8 GB).

In summary, PERGA generated better results than other assemblers for both the simulated and real *S.pombe* datasets D5∼D6, which indicated that the SVM model and the look-ahead approach were suitable for the assembly of other genomes and resulted in good performance.

### Performance on human chromosome 14

To highlight the performance of PERGA, we used the human chromosome 14 simulated 50x dataset D7 and real 40x dataset D8 to test its performance. MaSuRCA could not be run correctly on the simulated dataset because of unknown running error, and the results for the assemblers were shown in Tables 10–11.

From Table 10, PERGA generated the least number of contigs with the largest contigs (N50 size 149.9 kbp, maximal size 1 Mbp, mean size 36 kbp) and largest scaffolds (N50 size 229.5 kbp, maximal size 1 Mbp, mean size 39 kbp) with fewer mis-assemblies than others. PERGA and IDBA-UD were the top two assemblers in scaffold N50 size (230 kbp and 174 kbp), maximal size (about 1 Mbp) and accuracy (mis-assembled scaffolds 30 kbp and 1.2 Mbp), whereas the scaffold N50 size and maximal size of others were <90 kbp and <500 kbp, respectively, and they generated more mis-assembled scaffolds (>5 Mbp) than PERGA and IDBA-UD. IDBA-UD was the fastest assembler and it generated much longer and more accurate results than ABySS and Velvet. Velvet, CABOG and ABySS generated the least accurate results and their contig sizes and scaffold sizes were also short, and the genome coverage of Velvet dropped greatly from 92.24% to 24.87% because of its lots of mis-assembled scaffolds (67 Mbp). SGA generated the most number (32077) of scaffolds with the shortest length (N50 size 5.5 kbp), and about 10 Mbp genome regions were missing, so its genome coverage was no more than 86%.

From Table 11, PERGA generated large contigs and scaffolds (N50 size 11.8 kbp and 20.2 kbp) with the high genome coverage (95.86% and 91.96%) and fewer mis-assemblies (2.8 Mbp and 6.1 Mbp), that is because PERGA tried to extend contigs in more accurate way. IDBA-UD, CABOG and PERGA were the top three assemblers in terms of N50 size (>10 kbp and >20 kbp for contigs and scaffolds, respectively) and maximal size (>100 kbp and >140 kbp for contigs and scaffolds), however, CABOG produced more errors (7.3 Mbp and 13 Mbp for contigs and scaffolds, respectively) than IDBA-UD and PERGA, and the N50 size of other assemblers were less than 7 kbp for contigs and scaffolds. MaSuRCA, ABySS and SGA generated the most accurate results; however, their lengths were short (scaffold N50 size <7 kbp), and also, the total summed assembly length of SGA was only 85% (75 Mbp) of the reference (88 Mbp), about 10 Mbp (10%) of genome regions were missing, which decreased its genome coverage (<85%). Velvet generated short (N50 size 3.8 kbp) but accurate contigs (error contigs 2.8 Mbp), however, its scaffolds were also short (6.6 kbp) and contained much more errors (21 Mbp), so its genome coverage dropped dramatically from 89% to 70%.

From the experiments on D1∼D8, PERGA performed faster than other assemblers because of several reasons. First, the *k*-mer hash table enabled the fast way of aligning reads to contigs while assembling. Second, reads from other genome regions with only a few bases aligned onto contigs were prevented from assembly, which reduced the computations of spurious overlap. Third, PERGA adopted the variable overlap size approach and used paired-end reads with the highest priority, which reduced the computations and speeded up the algorithm. Therefore, PERGA performed faster than other assemblers.

In summary, PERGA outperformed most of other assemblers when the genome sizes increase from small bacterial genomes (e.g. *E.coli*) to large human chromosomes (e.g. chromosome 14) in longer and more accurate assembly results, and IDBA-UD was the second best assembler and had similar performance, whereas other assemblers (e.g. Velvet, SGA, and ABySS) might be more suitable for small bacterial genomes rather than large genomes.

The experiments showed that the greedy-like prediction extension strategy has better performance than graph-based assemblers because it uses the SVM prediction model to eliminate sequencing errors, and uses the look-ahead approach to deal with sequencing errors and resolve short tandem repeats in genome, thus resulting longer and more accurate assembly results.

## Conclusions

In this article, we present PERGA, a novel *de novo* paired-end reads assembler, which can generate large and accurate assemblies using the greedy-like prediction strategy to handle branches and errors to give much better extensions. By using look-ahead approach, PERGA distinguishes sequencing errors and repeats accurately and separates different copies of short repeats to make the extension much longer and more accurate. Moreover, instead of using single-end reads to construct contigs, PERGA uses paired-end reads in the first step and gives different priority to different read overlap thresholds ranging from *O*_max_ to *O*_min_ to resolve the gap and branch problem. Experiments showed that PERGA could generate very long and accurate contigs and scaffolds with fewer mis-assembly errors both for simulated reads data and real data sets for both low and high coverage datasets than the existing methods on both small bacterial genomes (e.g., *E.coli* and *S.pombe*) and large complex genomes (e.g., human chromosome 14).

## Supporting Information

File S1 **Example of tandem repeats in human chromosome and detailed assembly results.** The reference region 58,287,977–58,288,418 (region size 442 bp) of human chromosome 14 consists of three complex repeats A, B and C, with A appears three times, B appears four times, C appears five times, and A contains B as sub-repeat, B contains C as sub-repeat. PERGA can correctly resolve this repeat region but others fail.
(DOC)

File S2 **The detailed view of resolving tandem repeats in human chromosome 14 by PERGA.**
(PDF)
