# Supplementary material for: PERGA: A Paired-End Read Guided De Novo Assembler for Extending Contigs Using SVM and Look Ahead Approach
Source: PLoS One. 2014 Dec 2;9(12):e114253. doi: 10.1371/journal.pone.0114253 (PMC4252104; doi:10.1371/journal.pone.0114253)
Supplement: File S1 — Example of tandem repeats in human chromosome and detailed assembly results. The reference region 58,287,977–58,288,418 (region size 442 bp) of human chromosome 14 consists of three complex repeats A, B and C, with A appears three times, B appears four times, C appears five times, and A contains B as sub-repeat, B contains C as sub-repeat. PERGA can correctly resolve this repeat region but others fail. (DOC) [file pone.0114253.s001.doc]

Supporting Information for

# PERGA: A Paired-End Read Guided *De Novo* Assembler for Extending Contigs Using SVM and Look Ahead Approach

Xiao Zhu^1^*, Henry C.M. Leung^2^*, Francis Y.L. Chin^2^, Siu Ming Yiu^2^, Guangri Quan^3^, Bo Liu^1^, Yadong Wang^1§^

^1^Center for Bioinformatics, School of Computer Science and Technology, Harbin Institute of Technology, Harbin, Heilongjiang, China

^2^Department of Computer Science, University of Hong Kong, Pokfulam Road, Hong Kong

^3^National Pilot School of Software, Harbin Institute of Technology, Weihai, Shandong, China

*These authors contributed equally to this work

^§^Corresponding author

Correspondence should be addressed to: Yadong Wang

Email: [ydwang@hit.edu.cn](mailto:ydwang@hit.edu.cn)

Telephone: +86-451-86413316

Fax: +86-451-86413316

Email addresses:

XZ: [zhuxiao.hit@gmail.com](mailto:zhuxiao.hit@gmail.com)

HCML: [cmleung2@cs.hku.hk](mailto:cmleung2@cs.hku.hk)

FYLC: [chin@cs.hku.hk](mailto:chin@cs.hku.hk)

SMY: [smyiu@cs.hku.hk](mailto:smyiu@cs.hku.hk)

GQ: [grquan@hit.edu.cn](mailto:grquan@hit.edu.cn)

BL: [bo.liu@hit.edu.cn](mailto:bo.liu@hit.edu.cn)

YW: [ydwang@hit.edu.cn](mailto:ydwang@hit.edu.cn)

# 1. Resolve complex short tandem repeats

PERGA can resolve complex short tandem repeats. For example, the reference region 58,287,977-58,288,418 (region size 442 bp) of human chromosome 14 consists of three complex repeats A, B and C, with A appears three times, B appears four times, C appears five times, and A contains B as sub-repeat, B contains C as sub-repeat (Supporting Information S2). PERGA can resolve these complex repeats correctly but others cannot.

PERGA dealt with these repeats by resolving the branches one by one when extending contig. Firstly, PERGA resolved the branch caused by repeat A at reference position 58,288,418, and two copies were separated and put to their correct places. Note that reads from the third copy of repeat A could not be aligned to contig at the branch, so the copy was not detected. Secondly, PERGA resolved the second branch due to repeat B at reference position 58,288,237, and two copies were detected and put to their correct positions. Similarly, reads from other copies of repeat B could not be aligned to contig, so their repeat copies were not detected.

Thirdly, PERGA resolved the third branch due to repeat C at reference position 58,288,157, three copies were detected, and two of them had short distance of their paired-end reads, so they were invalid and were removed from that branch. And, the two copies were placed to their correct positions after the branch at reference position 58,288,026 were resolved.

Finally, all the repeats are correctly resolved by PERGA after dealing with the four branches caused by these repeats. Other assemblers could not correctly resolve these complex repeats, and they usually produced some short broken pieces instead.

# 2. Supporting Tables

## Table S1. Evaluation for *E.coli* simulated short reads data (D1, 50×).

|  |  | **Contigs** | | | | | | |  | **Scaffolds** | | | | | | |  |  |
| --- | --- | --- | --- | --- | --- | --- | --- | --- | --- | --- | --- | --- | --- | --- | --- | --- | --- | --- |
|  | ***k*/*O*** | **#ctgs** | **N50 (kbp)** | **Max. (kbp)** | **Sum (Mbp)** | **Mean (kbp)** | **Cov. (%)** | **Misass. (#/sum)** |  | **#scfs** | **N50 (kbp)** | **Max. (kbp)** | **Sum (Mbp)** | **Mean (kbp)** | **Cov. (%)** | **Misass. (#/sum)** | **Time (min)** | **Mem. (GB)** |
| PERGA | *O*≥25 | 81 | **174.7** | 327.3 | 4.58 | 56.5 | **100.0** | **0** |  | 78 | **174.7** | 327.3 | 4.58 | 58.7 | **100.0** | **0** | **3** | 0.9 |
| IDBA-UD | default | 153 | 112.6 | 327.1 | 4.58 | 29.9 | 99.98 | 2/559 |  | 119 | 148.5 | 327.1 | 4.58 | 38.5 | 99.98 | 1/321 | 11 | **0.6** |
| ABySS | *k*=45 | 110 | 119.2 | 270.3 | **4.64** | 42.2 | 99.90 | **0** |  | 103 | 119.2 | 270.3 | **4.64** | 45.1 | 99.42 | 1/3617 | 9 | 1.0 |
| Velvet | *k*=45 | 177 | 108.1 | 326.9 | 4.57 | 25.8 | 99.76 | 7/6658 |  | 153 | 148.3 | 326.9 | 4.57 | 29.9 | 99.89 | 1/1596 | **3** | 0.9 |
| SGA | *O*≥31 | 415 | 24.1 | 138.3 | 4.55 | 11.0 | 98.57 | **0** |  | 139 | 95.5 | 269.7 | 4.55 | 32.7 | 98.59 | 1/4120 | 43 | **0.6** |
| CABOG | default | 134 | 83.1 | 201.6 | 4.57 | 34.1 | 99.03 | 1/2638 |  | 98 | 88.5 | 204.0 | 4.57 | 46.7 | 99.03 | 1/2638 | 77 | 2.6 |
| MaSuRCA | default | **74** | 172.8 | **483.3** | **4.64** | **62.7** | 87.98 | 4/560k |  | **71** | 172.8 | **483.3** | **4.64** | **65.3** | 87.98 | 4/560k | 16 | 2.2 |

## Table S2. Evaluation for *E.coli* simulated short reads data (D2, 60×).

|  |  | **Contigs** | | | | | | |  | **Scaffolds** | | | | | | |  |  |
| --- | --- | --- | --- | --- | --- | --- | --- | --- | --- | --- | --- | --- | --- | --- | --- | --- | --- | --- |
|  | ***k*/*O*** | **#ctgs** | **N50 (kbp)** | **Max. (kbp)** | **Sum (Mbp)** | **Mean (kbp)** | **Cov. (%)** | **Misass. (#/sum)** |  | **#scfs** | **N50 (kbp)** | **Max. (kbp)** | **Sum (Mbp)** | **Mean (kbp)** | **Cov. (%)** | **Misass. (#/sum)** | **Time (min)** | **Mem. (GB)** |
| PERGA | *O*≥25 | 99 | **173.9** | 327.9 | 4.59 | 46.3 | **99.99** | **0** |  | 96 | **173.9** | 327.9 | 4.59 | 47.8 | **99.99** | **0** | **3** | 1.0 |
| IDBA-UD | default | 155 | 124.6 | 327.1 | 4.58 | 29.5 | **99.99** | **0** |  | 120 | **173.9** | 327.1 | 4.58 | 38.1 | 99.97 | **0** | 13 | **0.6** |
| ABySS | *k*=45 | 104 | 119.2 | 328.1 | **4.64** | 44.7 | 99.92 | **0** |  | 93 | 135.0 | 328.1 | **4.64** | 49.9 | 99.56 | 1/25k | 10 | 1.1 |
| Velvet | *k*=45 | 176 | 125.2 | 326.8 | 4.57 | 25.9 | 99.79 | 6/4451 |  | 155 | 148.5 | 326.8 | 4.57 | 29.1 | 99.87 | **0** | 5 | 1.0 |
| SGA | *O*≥31 | 421 | 23.5 | 138.2 | 4.55 | 10.8 | 98.35 | **0** |  | 137 | 95.4 | 269.5 | 4.54 | 33.2 | 98.48 | 1/492 | 50 | **0.6** |
| CABOG | default | 155 | 68.4 | 180.3 | 4.55 | 29.3 | 98.72 | **0** |  | 112 | 77.1 | 180.3 | 4.56 | 40.7 | 98.64 | 1/4996 | 98 | 2.6 |
| MaSuRCA | default | **70** | 156.4 | **328.4** | 4.63 | **66.2** | 94.25 | 2/257k |  | **69** | 156.4 | **328.4** | 4.63 | **67.2** | 94.25 | 2/257k | 19 | 2.2 |

## Table S3. Evaluation for *E.coli* simulated short reads data (D3, 100×).

|  |  | **Contigs** | | | | | | |  | **Scaffolds** | | | | | | |  |  |
| --- | --- | --- | --- | --- | --- | --- | --- | --- | --- | --- | --- | --- | --- | --- | --- | --- | --- | --- |
|  | ***k*/*O*** | **#ctgs** | **N50 (kbp)** | **Max. (kbp)** | **Sum (Mbp)** | **Mean (kbp)** | **Cov. (%)** | **Misass. (#/sum)** |  | **#scfs** | **N50 (kbp)** | **Max. (kbp)** | **Sum (Mbp)** | **Mean (kbp)** | **Cov. (%)** | **Misass. (#/sum)** | **Time (min)** | **Mem. (GB)** |
| PERGA | *O*≥25 | **84** | **174.7** | **328.1** | 4.58 | **54.6** | **99.99** | **0** |  | 84 | **174.7** | **328.1** | 4.58 | 54.6 | 99.99 | **0** | **5** | 1.2 |
| IDBA-UD | default | 153 | 124.6 | 327.1 | 4.58 | 29.9 | **99.99** | **0** |  | 113 | 148.6 | 327.1 | 4.58 | 40.1 | 99.96 | 2/1723 | 21 | 0.7 |
| ABySS | *k*=45 | 106 | 126.2 | **328.1** | **4.64** | 43.8 | 99.90 | 1/524 |  | 95 | 135.0 | **328.1** | **4.64** | 48.9 | 90.89 | 4/206k | 16 | 1.7 |
| Velvet | *k*=45 | 178 | 117.5 | 327.0 | 4.57 | 25.7 | 99.75 | 9/7347 |  | 159 | 148.5 | 327.0 | 4.57 | 28.8 | **100.0** | **0** | 7 | 1.4 |
| SGA | *O*≥31 | 442 | 21.7 | 121.0 | 4.55 | 10.3 | 98.16 | **0** |  | 116 | 105.6 | 269.7 | 4.55 | 39.2 | 98.46 | 2/1024 | 103 | **0.6** |
| CABOG | default | 196 | 37.3 | 180.3 | 4.37 | 22.3 | 93.63 | 1/61k |  | 135 | 56.7 | 189.0 | 4.37 | 32.4 | 93.56 | 1/65k | 209 | 2.6 |
| MaSuRCA | default | 87 | 148.8 | **328.1** | 4.65 | 53.4 | 98.89 | 2/54k |  | **83** | 172.2 | **328.1** | 4.65 | **56.0** | 92.15 | 4/371k | 29 | 2.4 |

## Table S4. Evaluation for *E.coli* real short reads data (D4, 600×).

|  |  | **Contigs** | | | | | | |  | **Scaffolds** | | | | | | |  |  |
| --- | --- | --- | --- | --- | --- | --- | --- | --- | --- | --- | --- | --- | --- | --- | --- | --- | --- | --- |
|  | ***k*/*O*** | **#ctgs** | **N50 (kbp)** | **Max. (kbp)** | **Sum (Mbp)** | **Mean (kbp)** | **Cov. (%)** | **Misass. (#/sum)** |  | **#scfs** | **N50 (kbp)** | **Max. (kbp)** | **Sum (Mbp)** | **Mean (kbp)** | **Cov. (%)** | **Misass. (#/sum)** | **Time (min)** | **Mem. (GB)** |
| PERGA | *O*≥25 | **115** | **133.5** | **316.8** | 4.58 | **40.0** | **99.99** | 1/207 |  | **105** | **154.8** | **316.9** | 4.58 | **43.7** | **99.99** | 1/207 | **21** | 3.8 |
| IDBA-UD | default | 144 | 106.8 | 236.6 | 4.57 | 31.8 | 99.93 | 1/2105 |  | 107 | 148.5 | 284.4 | 4.58 | 41.0 | 99.98 | **0** | 31 | 2.0 |
| ABySS | *k*=45 | 156 | 96.0 | 210.8 | **4.62** | 29.6 | 93.61 | 4/293k |  | 141 | 113.4 | 236.4 | **4.62** | 32.8 | 91.45 | 5/372k | 64 | **0.3** |
| Velvet | *k*=45 | 215 | 82.8 | 177.8 | 4.56 | 21.2 | 95.25 | 11/212k |  | 182 | 95.5 | 209.4 | 4.56 | 25.1 | 86.21 | 5/633k | 33 | 5.1 |
| SGA | *O*≥31 | 473 | 19.3 | 73.1 | 4.54 | 9.6 | 98.06 | **0** |  | 471 | 21.3 | 73.1 | 4.55 | 9.7 | 98.15 | 1/411 | 357 | 5.9 |
| CABOG | Could not be run correctly as it required lots of disk space that exceeded our machine | | | | | | | | | | | | | | | | | |
| MaSuRCA | default | 241 | 72.3 | 210.8 | 4.74 | 19.7 | 97.33 | 6/126k |  | 230 | 77.6 | 210.8 | 4.74 | 20.6 | 97.29 | 7/129k | 118 | 2.5 |

## Table S5. Evaluation for *S.pombe* simulated short reads data (D5, 50×).

|  |  | **Contigs** | | | | | | |  | **Scaffolds** | | | | | | |  |  |
| --- | --- | --- | --- | --- | --- | --- | --- | --- | --- | --- | --- | --- | --- | --- | --- | --- | --- | --- |
|  | ***k*/*O*** | **#ctgs** | **N50 (kbp)** | **Max. (kbp)** | **Sum (Mbp)** | **Mean (kbp)** | **Cov. (%)** | **Misass. (#/sum)** |  | **#scfs** | **N50 (kbp)** | **Max. (kbp)** | **Sum (Mbp)** | **Mean**  **(kbp)** | **Cov. (%)** | **Misass. (#/sum)** | **Time (min)** | **Mem. (GB)** |
| PERGA | *O*≥25 | 286 | 255.4 | 929.4 | 12.3 | 42.9 | 99.91 | **0** |  | 254 | 386.7 | **1610.3** | 12.3 | 48.3 | **99.90** | **0** | **8** | 1.7 |
| IDBA-UD | default | 422 | 137.7 | 449.1 | 12.3 | 29.1 | **99.99** | 3/966 |  | 270 | 254.7 | 1308.5 | 12.3 | 45.4 | 99.08 | 6/100k | 31 | **1.3** |
| ABySS | *k*=45 | 328 | 181.8 | 784.3 | 12.4 | 37.7 | 99.78 | 12/9k |  | 270 | 211.0 | 935.4 | 12.4 | 45.9 | 76.72 | 21/2.7M | 25 | 1.6 |
| Velvet | *k*=45 | 496 | 158.6 | 680.5 | 12.2 | 28.4 | 99.74 | 15/6k |  | 431 | 293.2 | 1190.6 | 12.2 | 24.6 | 99.74 | 8/8k | 11 | 1.9 |
| SGA | *O*≥31 | 844 | 43.0 | 181.4 | 12.2 | 14.4 | 98.12 | 1/214 |  | 402 | 155.1 | 557.8 | 12.2 | 30.3 | 98.95 | 4/27k | 103 | 2.0 |
| CABOG | default | 295 | 139.6 | 414.1 | 12.1 | 41.1 | 95.24 | 3/218k |  | 217 | 157.1 | 418.5 | 12.1 | 55.9 | 90.88 | 6/778k | 243 | 2.5 |
| MaSuRCA | default | **171** | **417.9** | **1055** | **12.6** | **73.8** | 90.76 | 6/1.3M |  | **166** | **417.9** | 1055 | **12.6** | **76.0** | 90.76 | 6/1.3M | 43 | 2.7 |

## Table S6. Evaluation for *S.pombe* real short reads data (D6, 52×).

|  |  | **Contigs** | | | | | | |  | **Scaffolds** | | | | | | |  |  |
| --- | --- | --- | --- | --- | --- | --- | --- | --- | --- | --- | --- | --- | --- | --- | --- | --- | --- | --- |
|  | ***k*/*O*** | **#ctgs** | **N50 (kbp)** | **Max. (kbp)** | **Sum (Mbp)** | **Mean (kbp)** | **Cov. (%)** | **Misass. (#/sum)** |  | **#scfs** | **N50 (kbp)** | **Max. (kbp)** | **Sum (Mbp)** | **Mean**  **(kbp)** | **Cov. (%)** | **Misass. (#/sum)** | **Time (min)** | **Mem. (GB)** |
| PERGA | *O*≥25 | 746 | **37.0** | **120.3** | 12.2 | 16.4 | **98.97** | 17/71k |  | 493 | **70.3** | **334.7** | 12.2 | 24.8 | **98.97** | **17/73k** | 7 | 1.7 |
| IDBA-UD | default | 964 | 32.1 | 89.1 | 12.2 | 12.7 | 98.54 | 28/140k |  | 590 | 54.0 | 270.3 | 12.2 | 20.8 | 97.56 | 35/247k | 31 | 1.3 |
| ABySS | *k*=45 | 923 | 33.3 | 111.4 | **12.3** | 13.3 | 98.20 | 44/73k |  | 821 | 35.7 | 111.4 | **12.3** | 14.9 | 96.29 | 48/230k | 21 | **0.8** |
| Velvet | *k*=45 | 1056 | 28.7 | 89.8 | 12.2 | 11.5 | 97.36 | 26/175k |  | 810 | 42.3 | 111.6 | 12.2 | 15.1 | 95.86 | 29/391k | **6** | 3.9 |
| SGA | *O*≥31 | 1376 | 21.3 | 73.4 | 12.1 | 8.8 | 97.10 | **16/38k** |  | 806 | 39.1 | 118.8 | 12.1 | 15.1 | 97.32 | 21/100k | 114 | 2.0 |
| CABOG | default | 888 | 22.3 | 93.5 | 12.0 | 13.5 | 95.12 | 8/71k |  | **415** | 49.4 | 171.6 | 12.0 | **29.0** | 98.95 | 11/396k | 705 | 7.3 |
| MaSuRCA | default | **729** | 36.2 | 100.4 | **12.3** | **16.8** | 97.0 | 17/210k |  | 465 | 64.7 | 175.9 | **12.3** | 26.5 | 93.71 | 20/672k | 70 | 2.7 |

## Table S7. Evaluation for human chromosome 14 simulated short reads data (D7, 50×).

|  |  | **Contigs** | | | | | | |  | **Scaffolds** | | | | | | |  |  |
| --- | --- | --- | --- | --- | --- | --- | --- | --- | --- | --- | --- | --- | --- | --- | --- | --- | --- | --- |
|  | ***k*/*O*** | **#ctgs** | **N50 (kbp)** | **Max. (kbp)** | **Sum (Mbp)** | **Mean (kbp)** | **Cov. (%)** | **Misass. (#/sum)** |  | **#scfs** | **N50 (kbp)** | **Max. (kbp)** | **Sum (Mbp)** | **Mean (kbp)** | **Cov. (%)** | **Misass. (#/sum)** | **Time (min)** | **Mem. (GB)** |
| PERGA | *O*≥25 | **2382** | **149.9** | **1025** | 86.7 | **36.4** | 99.54 | 22/60k |  | **1947** | **229.5** | **1032** | 86.7 | 39.2 | **99.58** | **21/30k** | 169 | 9.3 |
| IDBA-UD | default | 5900 | 66.7 | 358.7 | **87.1** | 14.8 | **99.74** | 44/152k |  | 3571 | 174.3 | 980.6 | 87.0 | 24.4 | 98.51 | 58/1.24M | **144** | 8.7 |
| ABySS | *k*=45 | 29378 | 11.4 | 113.6 | 86.0 | 2.9 | 94.98 | 377/354k |  | 20369 | 30.2 | 287.8 | 87.0 | 4.3 | 83.40 | 1109/11M | 331 | **6.0** |
| Velvet | *k*=45 | 21882 | 8.6 | 74.9 | 84.7 | 3.9 | 92.24 | 1642/3.4M |  | 3493 | 78.6 | 442.1 | **88.5** | 25.3 | 24.87 | 1655/67M | 147 | 13.5 |
| SGA | *O*≥31 | 55837 | 2.7 | 29.7 | 76.0 | 1.4 | 85.36 | **146/43k** |  | 32077 | 5.5 | 62.3 | 76.0 | 2.4 | 80.10 | 1980/5M | 1360 | 17 |
| CABOG | default | 2858 | 69.3 | 359.8 | 85.5 | 29.9 | 76.43 | 285/19M |  | 2114 | 82.8 | 476.0 | 85.5 | **40.5** | 67.40 | 318/26.6M | 2742 | 11 |
| MaSuRCA | Could not be run correctly because of unknown running error | | | | | | | | | | | | | | | | | |

## Table S8. Evaluation for human chromosome 14 real short reads data (D8, 40×).

|  |  | **Contigs** | | | | | | |  | **Scaffolds** | | | | | | |  |  |
| --- | --- | --- | --- | --- | --- | --- | --- | --- | --- | --- | --- | --- | --- | --- | --- | --- | --- | --- |
|  | ***k*/*O*** | **#ctgs** | **N50 (kbp)** | **Max. (kbp)** | **Sum (Mbp)** | **Mean (kbp)** | **Cov. (%)** | **Misass. (#/sum)** |  | **#scfs** | **N50 (kbp)** | **Max. (kbp)** | **Sum (Mbp)** | **Mean (kbp)** | **Cov. (%)** | **Misass. (#/sum)** | **Time (min)** | **Mem. (GB)** |
| PERGA | *O*≥25 | 17221 | 11.8 | **133.3** | **86.8** | 5.1 | **95.86** | 435/2.8M |  | 13343 | 20.2 | **177.8** | **86.6** | 6.5 | 91.96 | 423/6.1M | 194 | 7.9 |
| IDBA-UD | default | 11267 | **16.3** | 112.1 | 86.3 | 7.6 | 94.81 | 351/4.1M |  | 9005 | **21.8** | 147.6 | 86.2 | 9.5 | 92.94 | 335/5.8M | 122 | 8.0 |
| ABySS | *k*=45 | 60054 | 3.9 | 40.5 | 84.9 | 1.4 | 92.06 | 485/574k |  | 58267 | 4.1 | 44.5 | 84.9 | 1.5 | 91.61 | 547/975k | 161 | 6.4 |
| Velvet | *k*=45 | 38990 | 3.8 | 38.6 | 81.6 | 2.1 | 89.62 | 1199/2.8M |  | 24536 | 6.6 | 63.3 | 82.8 | 3.3 | 70.44 | 4342/21M | **68** | 6.5 |
| SGA | *O*≥31 | 57883 | 2.4 | 28.0 | 75.2 | 1.3 | 84.36 | **255/95k** |  | 53197 | 2.7 | 28.0 | 74.8 | 1.4 | 84.21 | 247/1.5M | 826 | 16 |
| CABOG | default | **9966** | 13.0 | 101.0 | 83.6 | **8.3** | 87.65 | 527/7.3M |  | **6582** | 20.6 | 141.9 | 83.6 | **12.7** | 82.07 | 560/13M | 1757 | 10 |
| MaSuRCA | default | 23154 | 6.8 | 60.5 | 84.7 | 3.7 | 95.38 | 165/616k |  | 22890 | 6.9 | 60.5 | 84.7 | 3.7 | **95.28** | **170/710k** | 478 | **2.7** |
