# Supplementary figures and images for: PERGA: A Paired-End Read Guided De Novo Assembler for Extending Contigs Using SVM and Look Ahead Approach
Source: PLoS One. 2014 Dec 2;9(12):e114253. doi: 10.1371/journal.pone.0114253 (PMC4252104; doi:10.1371/journal.pone.0114253)

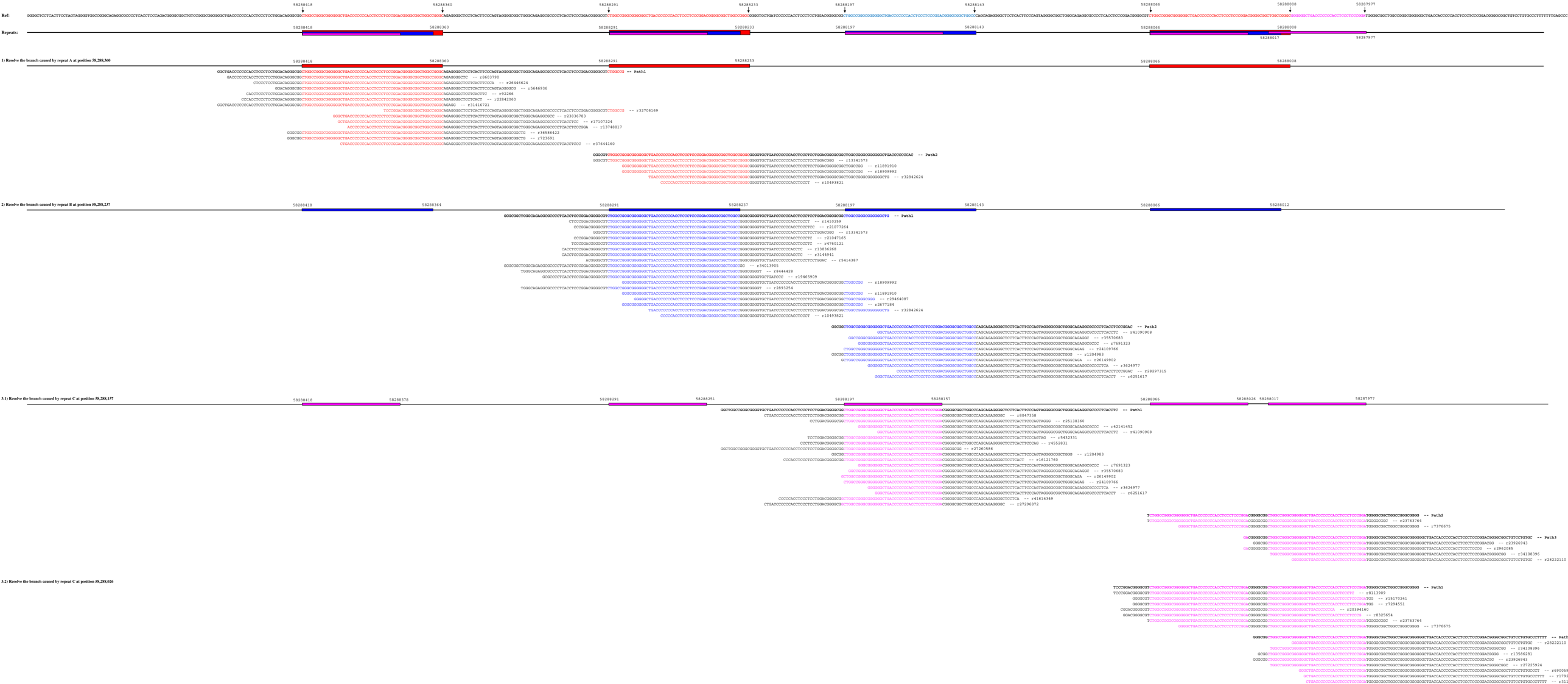

Supplement: File S2 — The detailed view of resolving tandem repeats in human chromosome 14 by PERGA. (PDF) [file pone.0114253.s002.pdf]
